# Enhancing Defect-Induced Dipole Polarization Strategy of SiC@MoO_3_ Nanocomposite Towards Electromagnetic Wave Absorption

**DOI:** 10.1007/s40820-024-01478-2

**Published:** 2024-08-16

**Authors:** Ting Wang, Wenxin Zhao, Yukun Miao, Anguo Cui, Chuanhui Gao, Chang Wang, Liying Yuan, Zhongning Tian, Alan Meng, Zhenjiang Li, Meng Zhang

**Affiliations:** 1 College of Chemical Engineering, Qingdao University of Science and Technology, Qingdao, 266042 People’s Republic of China; 2 College of Materials Science and Engineering, Qingdao University of Science and Technology, Qingdao, 266042 People’s Republic of China; 3 Shandong Engineering Laboratory for Preparation and Application of High-Performance Carbon-Materials, College of Electromechanical Engineering, Qingdao University of Science and Technology, Qingdao, 266061 People’s Republic of China; 4 Key Laboratory of Optic-Electric Sensing and Analytical Chemistry for Life Science, MOE, Shandong Key Laboratory of Biochemical Analysis, College of Chemistry and Molecular Engineering, Qingdao University of Science and Technology, Qingdao, 266042 People’s Republic of China

**Keywords:** Defect engineering, Oxygen vacancies, SiC@MoO_3_ nanocomposite, Electromagnetic wave absorption, Induced dipole polarization

## Abstract

Defect engineering in transition metal oxides semiconductors (TMOs) is attracting considerable interest due to its potential to enhance conductivity by intentionally introducing defects that modulate the electronic structures of the materials. However, achieving a comprehensive understanding of the relationship between micro-structures and electromagnetic wave absorption capabilities remains elusive, posing a substantial challenge to the advancement of TMOs absorbers. The current research describes a process for the deposition of a MoO_3_ layer onto SiC nanowires, achieved via electro-deposition followed by high-temperature calcination. Subsequently, intentional creation of oxygen vacancies within the MoO_3_ layer was carried out, facilitating the precise adjustment of electromagnetic properties to enhance the microwave absorption performance of the material. Remarkably, the SiC@MO-t4 sample exhibited an excellent minimum reflection loss of − 50.49 dB at a matching thickness of 1.27 mm. Furthermore, the SiC@MO-t6 sample exhibited an effective absorption bandwidth of 8.72 GHz with a thickness of 2.81 mm, comprehensively covering the entire Ku band. These results not only highlight the pivotal role of defect engineering in the nuanced adjustment of electromagnetic properties but also provide valuable insight for the application of defect engineering methods in broadening the spectrum of electromagnetic wave absor ption effectiveness. SiC@MO-t samples with varying concentrations of oxygen vacancies were prepared through in-situ etching of the SiC@MoO_3_ nanocomposite. The presence of oxygen vacancies plays a crucial role in adjusting the band gap and local electron distribution, which in turn enhances conductivity loss and induced polarization loss capacity. This finding reveals a novel strategy for improving the absorption properties of electromagnetic waves through defect engineering.

**Supplementary Information:**

The online version contains supplementary material available at 10.1007/s40820-024-01478-2.

## Highlights

- Oxygen-vacancy-rich SiC@MoO_3_ nanocomposite with strong reflection loss (− 50.49 dB at 1.27 mm thickness) and broadband absorption band (8.72 GHz at 2.68 mm thickness) were constructed via in situ etching strategy.
- The presence of oxygen vacancy leads to an increased conductive loss and defect-induced dipole polarization, which plays significant role in attenuating the incident electromagnetic wave.

**Supplementary Information:**

The online version contains supplementary material available at 10.1007/s40820-024-01478-2.

## Graphical abstract

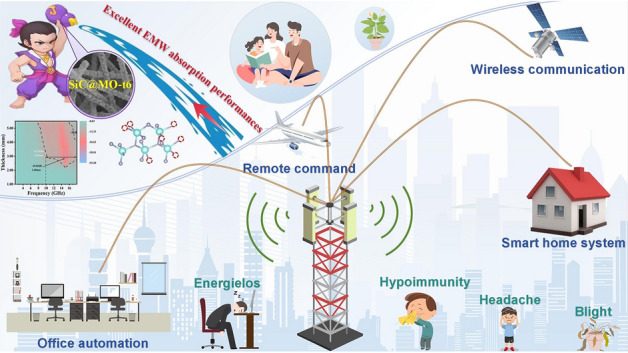

## Introduction

With the advancement of technological innovation, wireless communication facilitated by electromagnetic wave has significantly reduced the spatial distances among individuals. Simultaneously, the widespread integration of smart appliances and advanced office automation equipment is progressively reshaping societal habits. However, the ongoing degradation of the electromagnetic environment not only disrupts the reliable operation of precision instruments but also risks causing irreversible harm to human health [1–3]. Consequently, scholars universally agree on the urgent need to develop innovative electromagnetic wave-absorbing materials that are highly performant, easily manufacturable, and environmentally sustainable, with the goal of effectively mitigating the harmful impact of electromagnetic waves [4, 5]. To date, a diverse array of electromagnetic wave-absorbing materials has emerged, encompassing carbon-based substances, ceramics, metal composites, conductive polymers, and meta-materials [6–8].

Transition metal oxides (TMOs) are recognized as conventional dielectric loss absorbers that can effectively attenuate incident electromagnetic waves over a broad frequency range through conductive loss and dielectric polarization, thereby achieving better performances than magnetic loss absorbers [9, 10]. Among them, molybdenum oxide (MoO_3_), noted for its distinctive layered structure, low density, wide availability of raw materials, ease of preparation, corrosion resistance, and environmental compatibility, has emerged as a prominent member of the dielectric loss absorber family. However, bare MoO_3_ often exhibits suboptimal electrical conductivity, limited relaxation polarization channels, and poor impedance matching. These shortcomings lead to weak reflection loss, a narrow effective absorption bandwidth, and undesirable matching thickness. These limitations have persistently resulted in inferior electromagnetic wave absorption performance, impeding its broader application [11–13].

For dielectric loss absorbers, interfacial polarization, dipole polarization, and relaxation polarization play significant roles in the attenuation of electromagnetic waves. Research has revealed that employing materials with high electrical conductivity as substrates/carrier and combining them with MoO_3_ to fabricate MoO_3_-based nanocomposite absorbers enhances not only their electrical properties but also leverages the interfacial polarization at the composite interface [14–16]. This methodology significantly enhances the complex permittivity of the absorber, thereby significantly increasing the attenuation of electromagnetic waves and consequently yielding an absorption performance superior to that of single-component absorbers.

Building upon the previously outlined research strategy, researchers have already attained some valuable results in the study of the electromagnetic wave absorption performance of MoO_3_-based nanocomposite. However, several critical challenges persist, mainly including (1) The proportion of the biphase interface area on the surfaces of MoO_3_ and the substrate components is exceedingly small, resulting in a limited interfacial polarization effect. This fails to fully exploit the advantage of the high specific surface area of nanomaterials. (2) The strategies adopted to enhance electromagnetic wave absorption performance are consistent with those used for non-semiconducting transition metal oxides, neglecting the semiconducting nature of MoO_3_ and not intervening in the electron transport process. (3) The attenuation effect of oxygen vacancies on electromagnetic wave is commonly ascribed to induced dipole polarization, yet there is a lack of microscopic understanding and investigation of the fundamental mechanisms underlying electromagnetic attenuation.

Currently, the induction of oxygen vacancies has become one of the prevalent methods for modulating the physical properties of semiconductor nanomaterials [17, 18]. Research has shown that the introduction of oxygen vacancies can create defect states within the band gap, which alters the band edges and band gap structure, providing additional free electrons that increase electrical conductivity. Additionally, this can affect the magnetic ordering and exchange interactions of intrinsic products, and also enhance photocatalytic activity by trapping charge carriers and reducing recombination rates [19–22]. This consequently improves impedance matching and increases the dielectric loss of the materials in an alternating electromagnetic field, thereby attaining electromagnetic wave absorption performance that surpasses pristine samples.

In this study, MoO_3_, characterized as a wide-band gap semiconductor, served as the subject of research, beginning with the modulation of its electronic structure. Using SiC nanowires as a carrier, SiC@MoO_3_ nanocomposites were successfully synthesized via electro-deposition and calcination processes. Subsequently, through in-situ etching with KBH_4_, SiC@MO-t samples exhibiting diverse morphology and oxygen vacancy concentrations were prepared. By introducing oxygen vacancy defects into the intrinsic products, the conductive and induced dipole polarization loss effects associated with MoO_3_ upon incident electromagnetic waves were enhanced. This simultaneously improved the impedance matching of these products, resulting in improved electromagnetic wave absorption performance. Furthermore, in concert with insights from density functional theory calculations, a mechanism understanding of how oxygen vacancies contribute to the enhanced electromagnetic wave absorption performance of SiC@MoO_3_ nanocomposites has been proposed. This study provides a systematic framework for employing vacancy engineering to enhance the electromagnetic wave absorption properties of TMOs and paves the way for the development of efficient, scalable absorbing materials.

## Experimental Section

### Materials

The raw materials employed in this investigation were sodium hydroxide (NaOH, AR), anhydrous ethanol (C_2_H_5_OH, AR), ammonium molybdate tetrahydrate ((NH_4_)_6_Mo_7_O_24_·4H_2_O, AR), and potassium borohydride (KBH_4_, AR). All of these reagents were procured from China Pharmaceutical Group Chemical Reagents Co., LTD., and used without any additional purification.

### Preparation of SiC@MoO_3_ Nanocomposite

In this work, the substrate material was SiC nanowires, a product of our past research [23]. Initially, the SiC nanowires were immersed in a 3 M sodium hydroxide solution to eliminate the impurities on the surface. Subsequently, the residual impurities were removed by rinsing with deionized water followed by immersion to achieve neutrality for later use. Electro-deposition was carried out in a standard three-electrode configuration, with SiC nanowires serving as the working electrode, Pt wires and a saturated calomel electrode (SCE) were employed as the counter electrode and reference electrode, respectively. MoO_3_ deposition on SiC nanowires proceeded from an aqueous electrolyte solution of 15 mmol L^−1^ (NH_4_)_6_Mo_7_O_24_·4H_2_O using a CHI660e (Chenhua Instrument Co., Shanghai, China) electrochemical workstation operating at − 10 mA cm^−2^ for 400 s. The SiC@MoO_3_ nanocomposite was then obtained through calcination at 350 °C for 90 min under air at a ramp rate of 2 °C min^−1^.

### Preparation of Various SiC@MO-t Samples

**Fig. 1:**
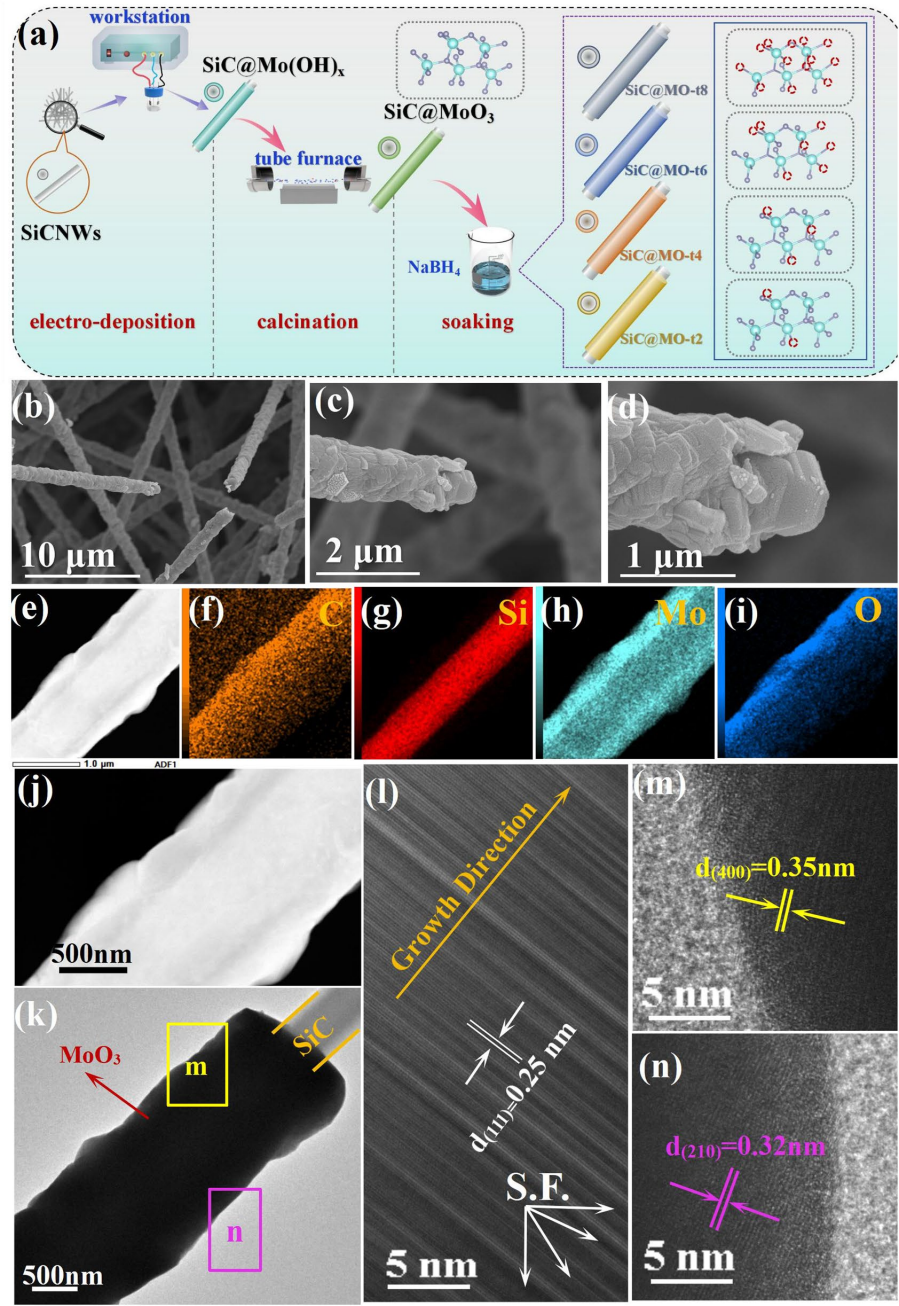
**a** Schematic illustration of SiC@MoO_3_ nanocomposite and SiC@MO-t samples synthesized at different KBH_4_ soaking time. **b–d** SEM images of SiC@MoO_3_ nanocomposite. **e–i** C, Si, Mo, and O element mappings of SiC@MoO_3_ nanocomposite. **j** Content of different elements in SiC@MoO_3_ nanocomposite. **k–n** Representative TEM and HRTEM images of SiC@MoO_3_ nanocomposite

The as-prepared SiC@MoO_3_ nanocomposites were immersed in a 2 M KBH_4_ aqueous solution for 2, 4, 6, and 8 min, respectively, resulting in the acquisition of nanocomposites with different oxygen vacancy concentrations. These products were denoted as SiC@MO-t2, SiC@MO-t4, SiC@MO-t6, and SiC@MO-t8, respectively. Figure 1a depicts the preparation processes of SiC@MoO_3_ nanocomposites and various SiC@MO-t samples, where the red dotted rings in the ball-and-stick model indicate different concentrations of oxygen vacancies.

### Characterization

The morphology of the nanocomposites was examined using a scanning electron microscope (SEM, Hitachi S-4800), while the micro-structure was verified using transmission electron microscopy (TEM, Hitachi H-8100). The phase composition of the SiC@MoO_3_ nanocomposite and SiC@MO-t samples was examined using a Rigaku D/max-2400 powder X-ray diffractometer (XRD). The chemical bonding and surface valence states were analyzed using X-ray Photo-electron Spectroscopy (XPS, Thermo ESCALAB 250XI). Electron paramagnetic resonance (EPR) measurements were performed using a Germany Bruker EMX plus paramagnetic resonance spectrometer. The resistance was measured using a multifunctional digital four-probe tester (ST-2258C multifunction digital four-probe tester). To evaluate the electromagnetic wave absorption properties, the samples with a filling ratio of ~ 30 wt% were combined with paraffin wax and compressed into a small ring with an inner diameter of 3 mm, an outer diameter of 7 mm, and a thickness of 3.04 mm. The electromagnetic parameters were measured in the 2–18 GHz frequency range using a Vector Network Analyzer (Agilent, N5230A).

## Results and Discussion

### Chemical Structure and Morphology of Oxygen Vacancy-Rich SiC@MoO_3_ Nanocomposites

SEM characterization was employed to examine the microstructure and morphology changes of the prepared products, and the corresponding results are shown in Figs. 1b–d and S1. Figure S1 displays SEM images of bare SiC nanowires, revealing that the surfaces of the nanowire are smooth, while their diameters exhibit bamboo-like periodic variations, with protruding nodules approximately 1 μm in diameter and constricted necks approximately 0.8 μm. Additionally, the aspect ratio of individual nanowires exceeds 20. In contrast to previously reported SiC nanowires with uniform diameters, the present structure features a higher specific surface area, offering a greater number of active sites for the growth of MoO_3_ [24]. Moreover, different SiC nanowires within the field of view intertwine randomly, forming a dense network structure. According to previous studies, the presence of this unique intertwined structure may enhance electron mobility and lead to significant conductive loss for incident electromagnetic waves [25–27]. Figure 1b–d presents representative SEM images of the SiC@MoO_3_ nanocomposite. The surface of the bare SiC nanowires is covered by flaky MoO_3_, with adjacent flakes overlapping at various angles, forming a thick shell. As depicted in Fig. 1d, the MoO_3_ flakes consist of numerous, orderly stacked nanosheets. Additionally, after successful modification with MoO_3_ flakes, the diameter of the nanowires increased to 1.1 μm, and their surfaces became significantly rougher, enhancing the potential for multiple reflection and scattering effects compared to those of smooth pure SiC nanowires [28].

Figure 1e–i illustrates the elemental mapping of the SiC@MoO_3_ nanocomposite. The elements C and Si are predominantly located in the central region, corresponding to the size and position of the SiC nanowire core. The elements Mo and O are primarily distributed on either side of the Si and C elements and originate predominantly from the MoO_3_ nanoshell. Figure S2 presents a representative EDS spectrum of the SiC@MoO_3_ nanocomposite. The components and their relative amounts identified in the spectrum closely correspond to those observed in the elemental mapping. Figure 1k presents the TEM image of a single SiC@MoO_3_ nanocomposite with a diameter of approximately 1 μm, a dimension consistent with the results shown in the SEM images of Fig. 1b. To further refine the lattice structure information of the product, HRTEM analyses were conducted on both the SiC nanowire core and the external MoO_3_ shell, with the results presented in Fig. 1l–n. Figure 1l illustrates distinct lattice fringes with a spacing of approximately 0.25 nm between adjacent crystal planes. This spacing is consistent with the cubic SiC (111) plane (JCPDS No. 29–1129), indicating that the SiC core is preferentially oriented along the [111] direction [29]. Additionally, some dark lattice fringes within the field of view have been noted, which can be attributed to stacking faults (S.F.) in the lattice according to previous studies [30]. Figure 1m, n presents the HRTEM images of the MoO_3_ shell, taken from the yellow and green square areas in Fig. 1k, respectively. The images show interplanar spacing of 0.35 and 0.32 nm, corresponding to the (400) and (210) planes of MoO_3_ [31]. These spacing indicate that the MoO_3_ grains exhibit varied crystal orientations across different thin film locations, demonstrating an overall poly-crystalline structure.

**Fig. 2:**
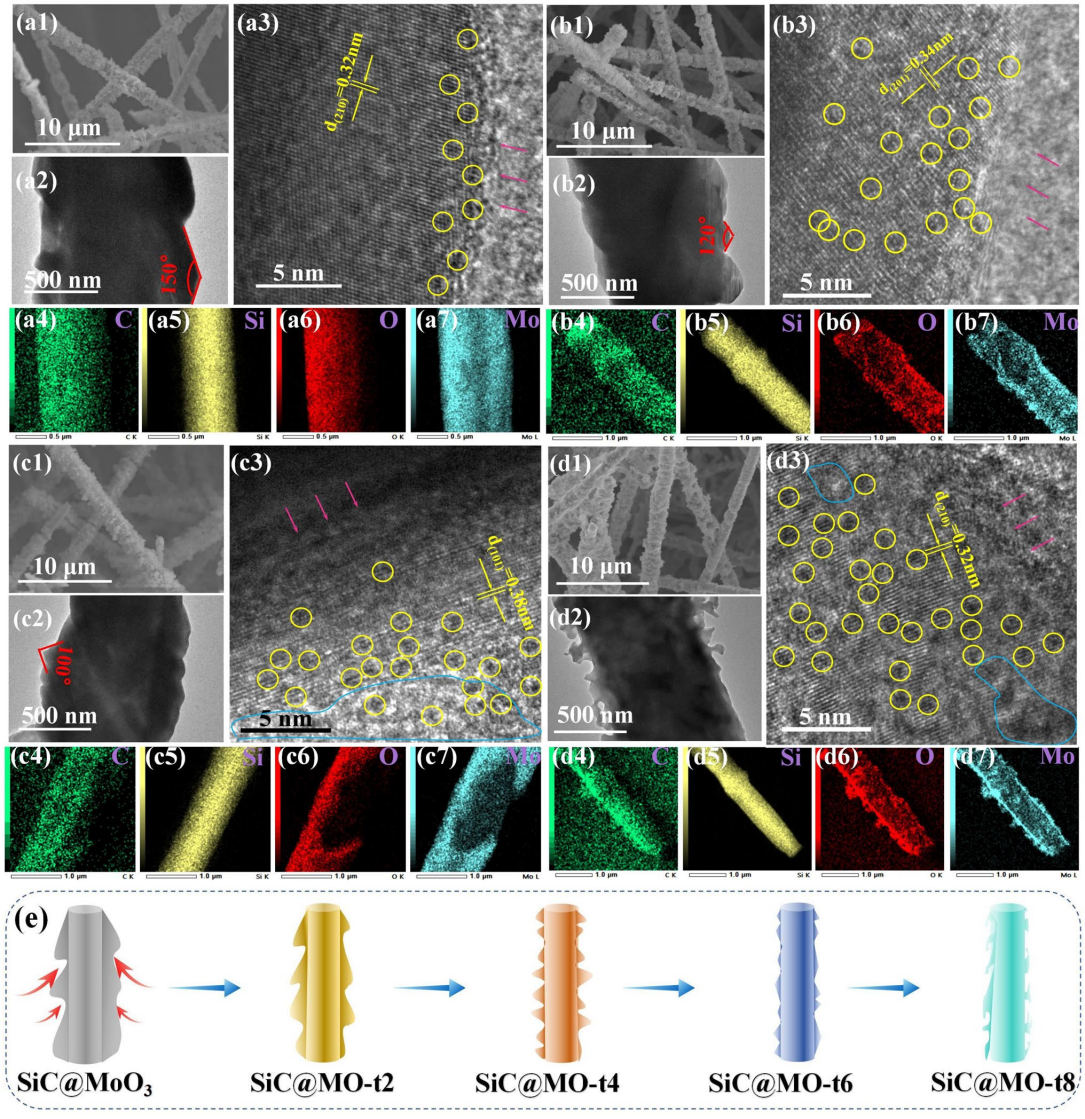
**a1–a7** SEM, TEM, HRTEM images and EDS mapping elements dispersion of SiC@MO-t2 nanocomposite. **b1–b7** SEM, TEM, HRTEM images and EDS mapping elements dispersion of SiC@MO-t4 nanocomposite. **c1–c7** SEM, TEM, HRTEM images and EDS mapping elements dispersion of SiC@MO-t6 nanocomposite. **d1–d7** SEM, TEM, HRTEM images and EDS mapping elements dispersion of SiC@MO-t8 nanocomposite. **e** Schematic illustration of the etching process of SiC@MO-t samples

Figure 2a–d depicts the SEM, TEM, HRTEM, and EDS mapping images of SiC@MoO_3_ nanocomposites fabricated under various KBH_4_ soaking time conditions. SEM characterization results demonstrate that after soaking treatment, various SiC@MO-t samples preserve their original linear morphology. However, compared to untreated samples, the surface of MoO_3_ nanosheets grows increasingly rougher, and the diameter of the nanowires progressively decrease as soaking time is extended. TEM images of samples prepared under various soaking times reveal that, due to the potent reducing action of KBH_4_, MoO_3_ nanosheet structures, initially hundreds of nanometers thick, are reduced to smaller, tens of nanometers thick.

The surface contour undergoes a gradual transition from 150 degrees (SiC@MO-t2, as depicted in Fig. 2a2) to 120 degrees (SiC@MO-t4, as illustrated in Fig. 2b2), and then to 100 degrees (SiC@MO-t6, as depicted in Fig. 2c2), ultimately leading to a coral-like morphology (SiC@MO-t8, as illustrated in Fig. 2d2), the schematic illustration was shown in Fig. 2e. Figure 2a3 presents a typical HRTEM image of the SiC@MO-t2 sample, displaying clear lattice fringes. The spacing between adjacent crystal planes is approximately 0.32 nm, corresponding to the (210) crystal plane of MoO_3_ [32]. At the edge of the crystalline area, very small dark spots are scattered (highlighted by yellow circles), likely resulting from the reduction of oxygen atoms in the MoO_3_ lattice by KBH_4_, resulting in oxygen vacancies. In Figs. 2b3–d3, besides the corresponding interplanar spacings that can be respectively labeled as the (201), (101), and (210) planes, oxygen vacancy defects similar to those in the SiC@MO-t2 sample can also be observed within the field of view. Additionally, as the soaking time increases, the generated oxygen vacancies gradually extend from the crystal surface to the crystal interior (as indicated by the pink arrows in the figures). Notably, in Fig. 2c3, d3, small amorphous regions appear within the MoO_3_ lattice (highlighted by blue curves), possibly due to the disordered arrangement of atomic structures resulting from a high concentration of oxygen vacancies [20, 33]. The O and Mo elemental distribution mappings of various samples exhibit phenomena consistent with the described changes in nanowire diameter and morphology.

**Fig. 3:**
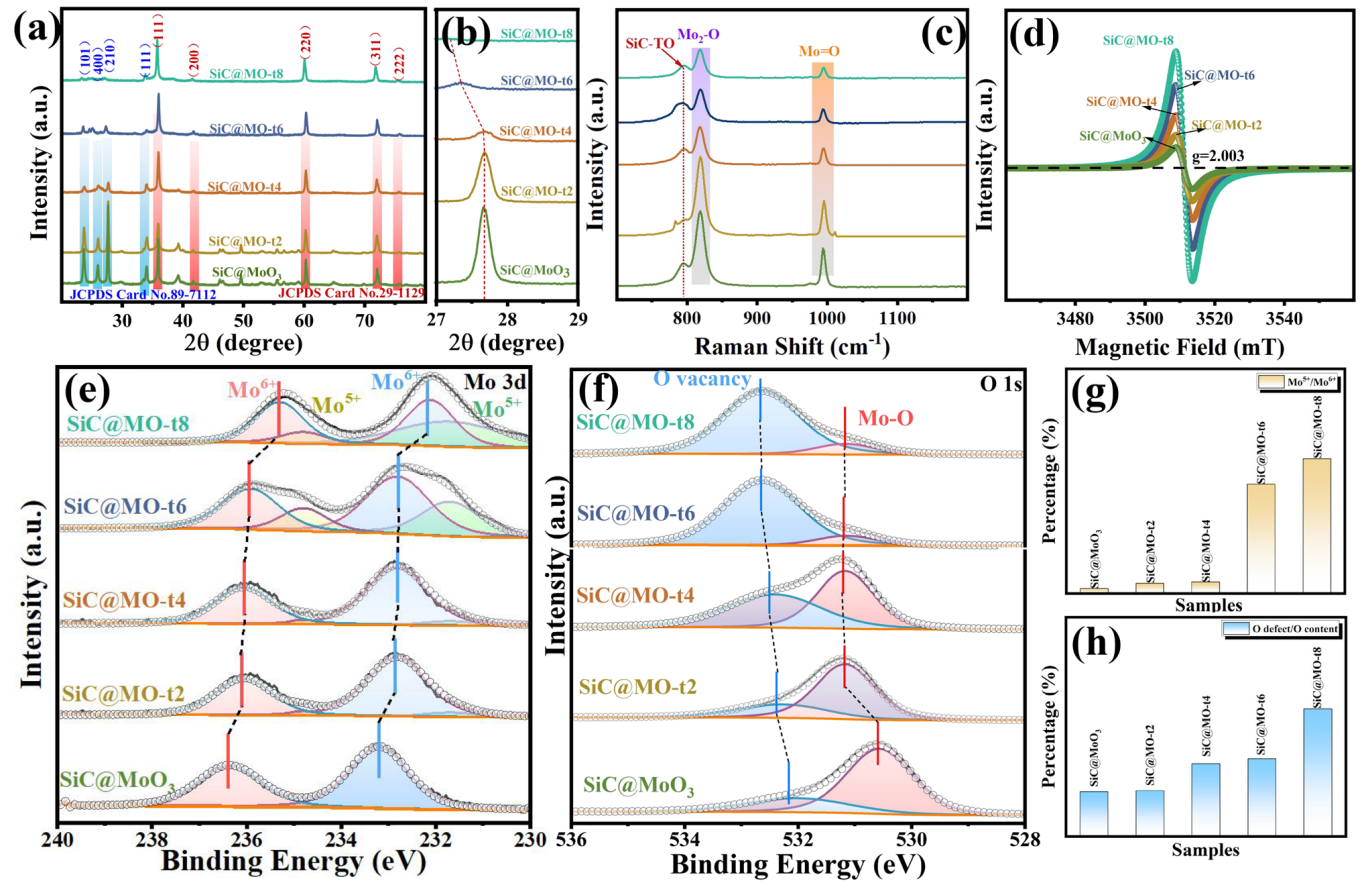
**a** XRD patterns of SiC@MoO_3_ nanocomposite and SiC@MO-t samples. **b** Partial enlarged detail of (210) diffraction peak. **c** Raman patterns of SiC@MoO_3_ nanocomposite and SiC@MO-t samples. XPS spectra of **d** O 1*s* and **e** Mo 3*d*. Ratio of the **f** Mo^5+^/Mo^6+^ and **g** O defect content in different samples. **h** EPR spectra of SiC@MoO_3_ nanocomposite and SiC@MO-t samples

Figure 3a displays the XRD patterns of the SiC@MoO_3_ nanocomposite and SiC@MO-t samples. Compared with the XRD pattern of bare SiC nanowires, the major diffraction peaks at 35.6°, 41.4°, 59.9°, 71.7°, and 75.5° are identified as the cubic SiC (JCPDS card No. 29–1129) planes (111), (200), (220), and (311), as marked by the orange arrows in the figure [29]. Furthermore, the diffraction peaks at 23.3°, 25.7°, 27.4°, and 33.7° are assigned to the MoO_3_ planes (101), (400), (210), and (111) (JCPDS card No. 89-7112), confirming the presence of MoO_3_ and aligning with HRTEM characterization results [31, 34]. Figure 3b presents an enlarged view of the (210) diffraction peak of MoO_3_, illustrating that as KBH_4_ soaking time is extended, the diffraction peak shifts noticeably towards smaller angles. According to the Bragg equation, this shift is likely associated with lattice distortions due to high concentrations of oxygen vacancies [35]. Noteworthily, the relative intensity of the diffraction peaks corresponding to MoO_3_ gradually decreases, while the intensity of the characteristic diffraction peaks corresponding to the SiC nanowires does not show significant changes across the samples. It can be reasonable speculated that this phenomenon may be related to the following two reasons: first, the partial etching of MoO_3_ when immersed in KBH_4_ leads to a reduction in coating thickness and a decrease in the relative content of MoO_3_ in the nanocomposite. Secondly, the introduction of oxygen vacancies into the MoO_3_ lattice induces changes in the intrinsic crystal structure, causing lattice distortion. In regions with a high concentration of oxygen vacancy defects, the disorderly arrangement of atoms is triggered, resulting in decreased crystallinity and weakened diffraction peaks. Figure 3c depicts the Raman spectra of the SiC@MoO_3_ nanocomposite and SiC@MO-t samples within the 600–1200 cm^−1^ range. The characteristic peak near 850 cm^−1^ corresponds to the TO mode of the SiC nanowire core, while the peak near 1000 cm^−1^ is potentially associated with the stretching vibrations of [MoO_6_] octahedral units comprising MoO_3_ [36, 37]. Compared to the SiC@MoO_3_ nanocomposite, soaking in the reducing agent KBH_4_ causes a shift of the Raman absorption peak associated with symmetric stretching vibrations towards lower wave-numbers. The longer the soaking time, the more pronounced the red-shift, indicating that the introduction of oxygen vacancies leads to a reduction in Mo^6+^ cations.

XPS spectroscopy was employed to study the chemical bonding information and surface valence state changes of the SiC@MoO_3_ nanocomposites and SiC@MO-t samples, with their corresponding total spectra are depicted in Fig. S3. Given the high stability of the SiC nanowires used, no significant changes were observed before and after soaking treatment. Therefore, only the O 1*s* and Mo 3*d* spectra were analyzed in detail, with the results displayed in Fig. 3d, e, respectively. The O 1*s* spectra derived from the SiC@MoO_3_ nanocomposite and SiC@MO-t samples typically exhibit two distinct fitted peaks, corresponding to lattice oxygen (Mo–O) at 530.5 eV and defect oxygen (oxygen vacancies) at 532.2 eV. Importantly, the defect oxygen characteristic peak shifts towards higher binding energies as the duration of the reducing agent treatment is extended, indicating a rise in the concentration of oxygen vacancies in the products [38, 39]. In Fig. 3d, only Mo^6+^ signals are observed in the SiC@MoO_3_ nanocomposite, with fitted peaks at 233.2 and 236.3 eV corresponding to Mo^6+^ 3*d*_5/2_ and 3*d*_3/2_, respectively [40, 41]. In the produced SiC@MO-t samples, the characteristic peaks of Mo^6+^ 3*d*_5/2_ and 3*d*_3/2_ shift towards lower binding energies, and characteristic signals of Mo^5+^ 3*d*_5/2_, and 3*d*_3/2_ emerge near 231.8 and 234.7 eV, potentially linked to the presence of oxygen vacancies in the lattice. To further explore the concentration of oxygen vacancies and the valence states of bonded atoms in different samples, the integral areas of various characteristic peaks were calculated, yielding the ratio of Mo^5+^/Mo^6+^ to the content of defect oxygen, as depicted in Fig. 3f. Clearly, as the soaking time increases, the content of Mo^5+^ and the concentration of oxygen vacancies increase. The ratio for SiC@MoO_3_ nanocomposite materials is very small (almost negligible). When the immersion time is short (2 and 4 min), the ratio of the integrated area of the Mo^5+^/Mo^6+^ characteristic peaks slightly increases in the SiC@MO-t2 and SiC@MO-t4 samples compared to the initial sample. However, with longer immersion times (6 and 8 min), the ratio of the integrated area of the Mo^5+^/Mo^6+^ characteristic peaks for the SiC@MO-t6 and SiC@MO-t8 samples rises sharply, reaching values several times higher than those of the SiC@MoO_3_ nanocomposite materials. Figure 3g is the integrated area ratio of the O defect peak relative to the combined area of the O defect and Mo–O peaks, exhibiting the same trend as in Fig. 3f, with the SiC@MO-t8 sample having the highest ratio. To verify and quantify the concentration of oxygen vacancies in SiC@MO-t samples, EPR testing was employed as a reference method. As depicted in Fig. 3h, the g-factor for all samples remains consistently at 2.003, largely because the oxygen vacancies in all samples capture electrons [42, 43]. The figure clearly illustrates that the EPR signal of SiC@MoO_3_ nanocomposite is significantly weak, suggesting a minimal presence of oxygen vacancies in the sample. With prolonged exposure to the reducing agent KBH_4_, the EPR signal of the SiC@MO-t samples increase in intensity, reflecting an upward trend in oxygen vacancy concentration, which corroborates the findings from earlier XRD and XPS analyses.

### Microwave Absorption Performance of Oxygen Vacancy-Rich SiC@MoO_3_ Nanocomposites

**Fig. 4:**
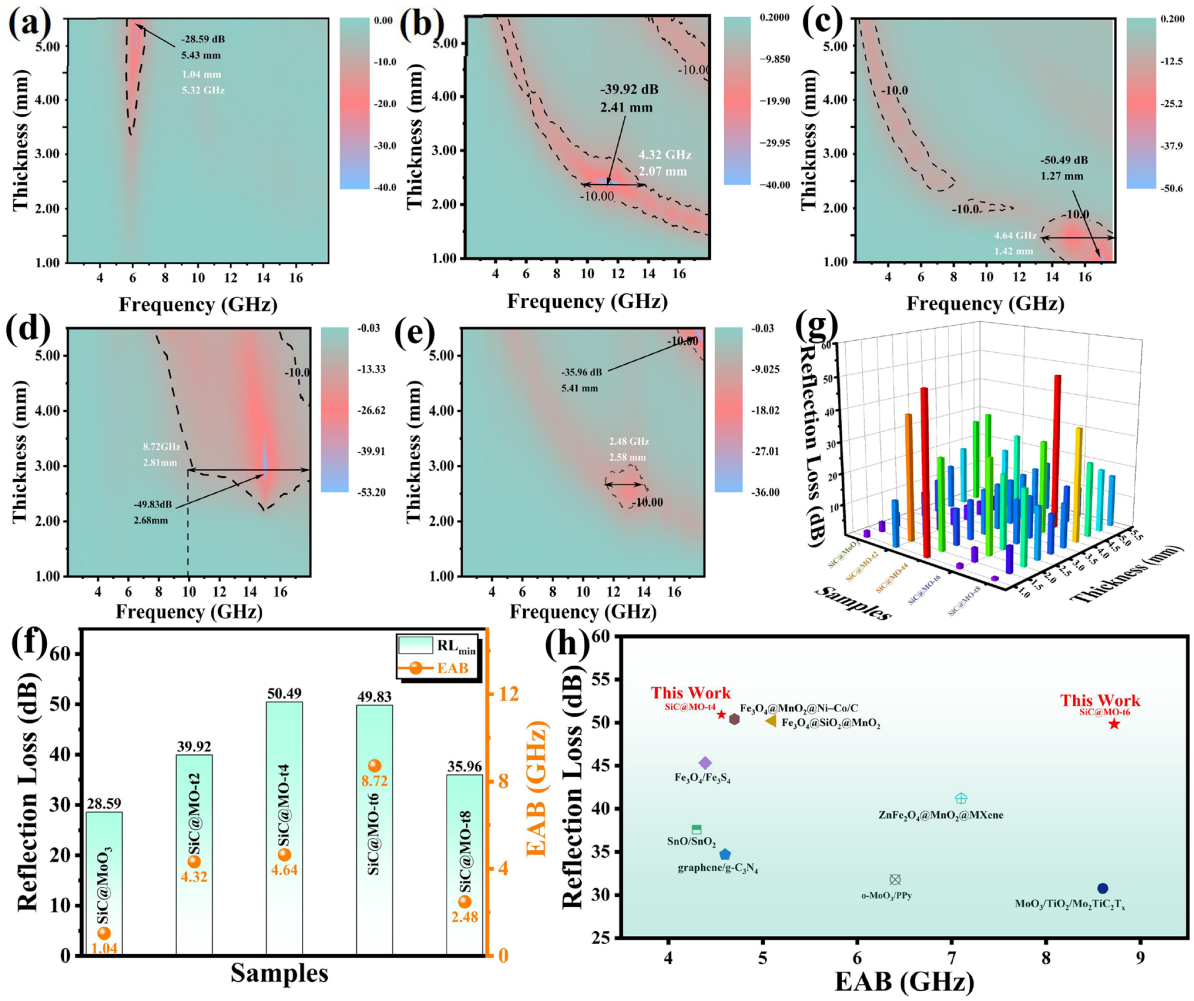
**a–e** RL values versus frequency of SiC@MoO_3_, SiC@MO-t2, SiC@MO-t4, SiC@MO-t6 and SiC@MO-t8 at different thickness. **f** Comparison of *RL*_min_ and EAB value of different samples at optimal matching thickness themselves. **g** *RL*_min_ comparison of different samples at various matching thickness. **h** Comparison of *RL*_min_ and EAB values of the SiC@MO-t4 and SiC@MO-t6 samples with recent reports related absorbers

The effectiveness of electromagnetic wave absorbing materials can be assessed by examining the relationship among thickness, electromagnetic wave frequency, and reflection loss (RL) values. The formula employed is as follows [44]:

1 $$ Z_{in} = Z_{0} \sqrt {\frac{{\mu_{r} }}{{\varepsilon_{r} }}} \tanh [j\left( {\frac{2\pi fd}{c}} \right)\sqrt {\mu_{r} \varepsilon_{r} ]} $$

2 $$\text{RL}(\text{dB})=20\text{log}\mid \frac{{\text{Z}}_{\text{in}}-{\text{Z}}_{0}}{{\text{Z}}_{\text{in}}+{\text{Z}}_{0}}\mid $$

where Z_in_ denotes the input impedance of the absorber, while Z_0_ denotes the impedance of free space. The relative complex permittivity and relative complex permeability are denoted by ε_r_ and μ_r_, respectively. Here, c denotes the speed of light, f denotes the frequency of the incident electromagnetic wave, and d denotes the thickness of the absorber. Figures S4 and S5 depict the relationship between the most pronounced minimum reflection loss (*RL*_min_) and effective absorption bandwidth (*EAB*) across varying matching thicknesses in SiC@MoO_3_ nanocomposite and SiC@MO-t samples, as functions of frequency. As depicted in the figures, each sample exhibits a significant frequency dispersion effect. Specifically, as the matching thickness increases, the *RL*_min_ values progressively transition from the high-frequency to the mid-low frequency range. Figure 4a–e presents two-dimensional projection drawings corresponding to Fig. S6. As demonstrated in Fig. 4a, the benchmark sample (SiC@MoO_3_ nanocomposite) achieves an optimal *RL*_min_ value of -28.59 dB within the C-band (approximately 6 GHz) and features a preferred EAB of 1.04 GHz, with corresponding matching thicknesses of 5.43 and 5.32 mm, respectively. As depicted in Fig. 4b, the SiC@MO-t2 sample achieves an optimal *RL*_min_ value of − 39.92 dB at a matching thickness of 2.41 mm and exhibits an optimal *EAB* value of 4.32 GHz at 2.07 mm matching thickness. At a matching thickness of 1.27 mm, the SiC@MO-t4 sample (Fig. 4c) exhibits the strongest *RL*_min_ value of -50.49 dB, with its optimal *EAB* value of 4.64 GHz occurring at a matching thickness of 1.42 mm. As shown in Fig. 4d, the SiC@MO-t6 sample displays the strongest *RL*_min_ value of -49.83 dB at a matching thickness of 2.68 mm. At a matching thickness of 2.81 mm, its EAB value achieves an impressive 8.72 GHz (9.28–18 GHz), encompassing the entire Ku band. For the SiC@MO-t8 sample (Fig. 4e), the optimal *RL*_min_ value is — 35.96 dB at a matching thickness of 5.41 mm, whereas the optimal EAB value is 2.48 GHz at a matching thickness of 2.58 mm. Previous reported work pointed out that the introduction of oxygen vacancies plays a critical role in modulating the frequency range over which electromagnetic wave attenuation occurs [45]. Compared to the SiC@MoO_3_ nanocomposite, the SiC@MO-t samples with increased oxygen vacancies exhibit a shift in their RL_min_ values and preferred EAB from lower frequency (C-band) to the mid-range (X band) or higher frequency (Ku-band). This shift is accompanied by reductions in their required matching thicknesses. These observations underscore the significance of oxygen vacancies in enhancing the electromagnetic wave absorbing performance of such materials. Obviously, the *RL*_min_ and *EAB* values gradually shift from high frequency to low frequency, which is in accordance with the 1/4 wavelength theory (as exhibited in Fig. S6). To further explore the absorption characteristics of the absorber, the relationship between the matching frequency (ƒ_*m*_) and the matching thickness (*t*_*m*_) was analyzed by the following equation.

3 $$ t_{m} = \frac{n\lambda }{4} = \frac{nc}{{4f_{m} \sqrt {\left| {\mu_{r} } \right|} \left| {\varepsilon_{r} } \right|}} \left( {n = 1,3,5.......} \right)\varepsilon_{r} $$

where *t*_*m*_ is the matching thickness of the absorber, λ is the wavelength of the incident electromagnetic wave, C represents the speed of light in vacuum, and ƒ_*m*_ is the corresponding matching frequency. It can be clearly seen that the perfect matching relationship between the thickness of the prepared series of samples and the propagation wavelength (λ/4, 3λ/4) is demonstrated, indicating that the quarter-wavelength theory effectively enables multi-band absorption performance. In comparison, SiC@MO-t4 and SiC@MO-t6 achieve optimal multi-band and multi-thickness effects, corresponding to excellent impedance matching capabilities of the material. They exhibit a wide range of exceptional matching performance in various bands and thicknesses. Figure 4f depicts the trends in *RL*_min_ and *EAB* values at the optimal matching thicknesses for various samples. Interestingly, it reveals that the enhancement in electromagnetic wave absorption performance due to oxygen vacancies does not exhibit a monotonically increasing trend. Instead, as the concentration of oxygen vacancies increases, the optimal values of *RL*_min_ and EAB values initially increase and subsequently decrease. To provide a more comprehensive and intuitive depiction of the electromagnetic wave absorption performance of each SiC@MO-t sample, bar charts illustrating the changes in *RL*_min_ and *EAB* values across various matching thicknesses were developed, as shown in Figs. 4g and S7. As indicated, both the SiC@MO-t4 and SiC@MO-t6 samples, possessing a moderate concentration of oxygen vacancies, demonstrate the highest *RL*_min_ and broadest *EAB* across multiple thicknesses, highlighting their superior absorption capabilities. Analysis of the XRD spectra and HRTEM images presented in Figs. 2 and 3 reveal that prolonged exposure to the reducing agent could result in an excessively high concentration of oxygen vacancies. This excess may disrupt the original MoO_3_ structure, consequently leading to suboptimal electromagnetic wave absorption performance in the SiC@MO-t8 sample compared to the SiC@MO-t6 sample. As previously discussed, while a small number of oxygen vacancies function as dipoles in an alternating electromagnetic field and exert limited effects, an excess of oxygen vacancies not only increases the concentration in local areas of the MoO_3_ shell but also significantly compromises its structural integrity. This results in an overall decrease in oxygen vacancy content within the shell. From both perspectives, there are limited improvements in the electromagnetic wave absorption performance of SiC@MoO_3_ nanocomposite. In contrast, both the SiC@MO-t4 and SiC@MO-t6 samples, which contain a moderate level of oxygen vacancies, strike an optimal balance between concentration and content, thereby significantly enhancing the electromagnetic wave absorption performance of the initial product. Figure 4i and Tab. S1 present a comparative analysis of the electromagnetic wave absorption performance of the SiC@MO-t4 and SiC@MO-t6 samples with recent reports on absorbers involving Mo and other oxides [46–53]. The results indicate that the synthesized SiC@MO-t4 and SiC@MO-t6 samples exhibit a wider *EAB* and more impressive *RL*_min_ values. These characteristics align with the ideal properties of absorptive materials, namely thinness, broad bandwidth, lightweight, and high efficiency.

**Fig. 5:**
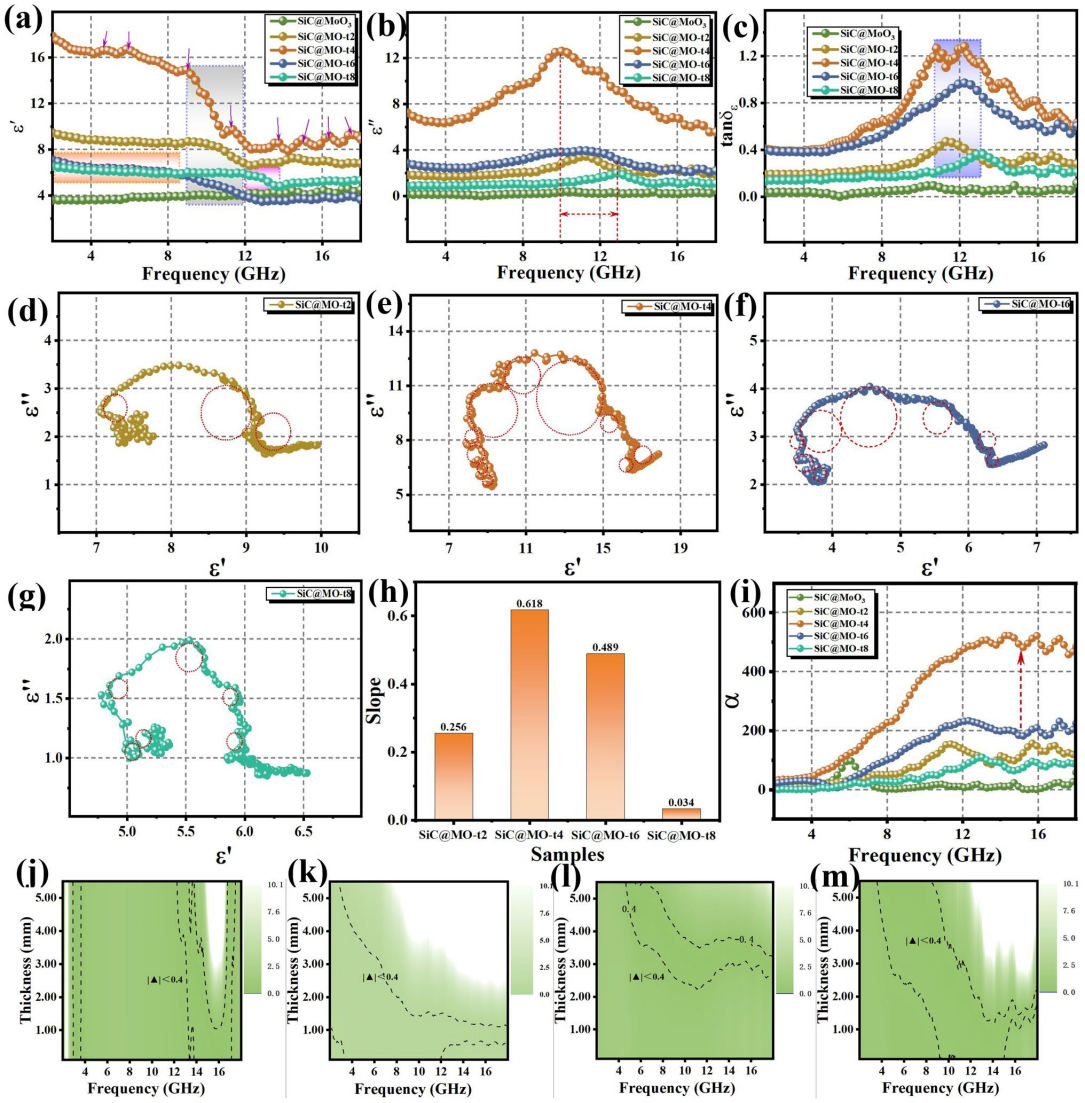
**a** Real part and **b** imaginary part of complex permittivity of different SiC@MO-t samples. **c** Dielectric loss tangent, **d–g** Cole–Cole curve of SiC@MO-t samples. **h** Trailing slop of Cole–Cole curve. **i** Attenuation constant of SiC@MoO_3_ nanocomposites and SiC@MO-t samples. **j–m** Delta value of SiC@MO-t samples

The complex permittivity (εr = ε′−jε′′) and complex permeability (μr = μ′−jμ′′) are crucial parameters in evaluating the performance of electromagnetic wave absorbing materials. The real parts (ε′ and μ′) and the imaginary parts (ε′′ and μ′′) respectively denote the storage and attenuation capacities of these materials for the electric and magnetic energy of incident electromagnetic waves [54]. These values are derived using the following formulas:

4 $$ \varepsilon_{r} = \varepsilon_{\infty } + \frac{{\varepsilon_{s} - \varepsilon_{\infty } }}{{1 + j2\pi f^{\prime}\tau }} = \varepsilon \prime - j\varepsilon \prime $$

5 $$ \varepsilon^{\prime} = \varepsilon_{\infty } + \frac{{\varepsilon_{s} - \varepsilon_{\infty } }}{{1 + (2\pi f)^{2} \tau^{2} }} $$

6 $$ \varepsilon^{\prime\prime} = \frac{{2\pi f\tau \left( {\varepsilon_{s} - \varepsilon_{\infty } } \right)}}{{1 + (2\pi f)^{2} \tau^{2} }} $$

herein, ε_∞_ represents the optical permittivity, ε_s_ indicates the static permittivity, τ signifies the relaxation time, and f denotes the frequency. Considering that both SiC nanowires and the MoO_3_ shell are non-magnetic materials, their real and imaginary parts of permeability values exhibit minor fluctuations around 1 and 0, respectively [26]. The nanocomposite demonstrates negligible magnetic loss to incident electromagnetic waves (Fig. S8), which can be reasonably disregarded in this analysis. This research focuses on the impact of dielectric loss on the attenuation of electromagnetic waves by these materials. Figure 5a, b depicts the frequency-dependent variations in ε′ and ε′′ values for the SiC@MoO_3_ nanocomposite and various SiC@MO-t samples, respectively. At low frequencies, the dielectric constant increases with increasing frequency because molecular polarization requires a certain amount of time. Conversely, at high frequencies, the dielectric constant decreases with increasing frequency because the relaxation time for molecular polarization is too short to complete polarization [55–57]. The ε′ and ε′′ values of the pure SiC@MoO_3_ nanocomposite remain stable at approximately 4 and 0.13, respectively. This stability may account for the lack of frequency-dependent variations in the *RL*_min_ values observed in Figs. 3a and S4. The remarkably low ε′′ value of the sample results in insubstantial dielectric loss to incident electromagnetic waves, posing challenges to achieving satisfactory wave absorption performance. Furthermore, a difference in values exceeding 20-fold indicates significant alterations to the impedance matching characteristics [58]. With the introduction of oxygen vacancies, the ε′ value of the SiC@MO-t samples rises markedly, indicating an enhanced capacity for electric field energy storage. Within the 2–9 GHz frequency range, the ε′ values of the samples remain stable, displaying smooth curves, with the SiC@MO-t4 sample exhibiting significantly higher values than the others. Meanwhile, the SiC@MO-t6 sample exhibits values comparable to those of SiC@MO-t8 sample in this frequency region, suggesting their indistinguishable electric field storage capability. In the frequency range of 9–12 GHz, most of the SiC@MO-t samples (SiC@MO-t2 ~ SiC@MO-t6) exhibit a gradually decrease in ε′ values. For the SiC@MO-t8 sample, the declining trend commences at approximately 12–14 GHz. Additionally, throughout the mid to high frequency range, all SiC@MO-t samples display multiple distinct resonance peaks, potentially linked to polarization effects induced by oxygen vacancies [2]. As illustrated in Fig. 5b, the ε′′ values of the SiC@MO-t samples initially increase and subsequently decrease. With an increase in the concentration of oxygen vacancies, the ε′′ values of the SiC@MO-t samples exhibit an overall trend of initially increasing and then decreasing. Notably, the SiC@MO-t4 sample demonstrates the highest ε′′ values compared to the other samples across the entire measured frequency range, indicating its superior attenuation capacity for the electric field energy of incident electromagnetic waves, with a peak value near 10 GHz, approximately 12.9 [59]. Throughout the entirety of the testing frequency range, the ε′′ values of the SiC@MO-t6 sample consistently range between 2 and 4, indicating its stable attenuation capacity for the electric field energy of incident electromagnetic waves. Additionally, the maximal ε′′ values of each SiC@MO-t sample are observed within either the X or Ku band, accompanied by multiple distinct resonance peaks. In Fig. 5a, the ε′ values of SiC@MO-t2 and SiC@MO-t8 are either slightly higher than or equivalent to those of the SiC@MO-t6 sample across the entire range of tested frequencies. However, in Fig. 5b, the values of the former two are consistently lower than those of the latter throughout the entire tested frequency range. This suggests a more pronounced disparity between their capabilities in storing and attenuating electric field energy, which may significantly influence the impedance matching characteristics of the materials and potentially have a detrimental effect on electromagnetic wave attenuation. Figure 5c presents the frequency-dependent curves of the loss tangent values (tanδ_ε_ = ε′′/ε′) for various products. Within the 2–10 GHz frequency range, a uniform trend is observed across all samples. The maximum tanδ_ε_ values for the SiC@MO-t samples with oxygen vacancies are observed within the 10–13 GHz frequency range, exhibiting a variation tendency similar to that of various samples exhibited in Fig. 5b. It is noteworthy that, in contrast to the results in Fig. 5b, the SiC@MO-t4 sample presents two distinct peaks in tanδ_ε_ values, and the disparity in tanδ_ε_ values between the SiC@MO-t4 and SiC@MO-t6 samples is notably reduced. These findings indicate that, in addition to ε′′, other parameters also significantly impact the electromagnetic wave attenuation characteristics.

Generally, the dielectric loss in wave-absorbing materials can be primarily attributed to conductive and polarization loss [60]. To elucidate the attenuation characteristics of incident electromagnetic waves on varied SiC@MO-t samples, resulting from oxygen vacancies, the values of conductive and polarization loss as functions of frequency were calculated using the prescribed formula. In this context, ε_p′′_ denotes polarization loss, ε_c′′_ denotes conductive loss, σ represents the conductivity, and ε_0_ (ε_0_ = 8.854 × 10^−12^ F m^−1^) represents the permittivity of free space [61].

7 $$ \varepsilon^{\prime\prime} = \varepsilon_{p}^{^{\prime\prime}} + \varepsilon_{c}^{^{\prime\prime}} = \left( {\varepsilon_{s} - \varepsilon_{\infty } } \right)\frac{2\pi f\tau }{{1 + \left( {2\pi f} \right)^{2} \tau^{2} }} + \frac{\sigma }{{2\pi f\varepsilon_{0} }} $$

8 $$ \varepsilon_{c}^{^{\prime\prime}} = \frac{\sigma }{{2\pi f\varepsilon_{0} }} $$

9 $$ \varepsilon_{p}^{^{\prime\prime}} = \left( {\varepsilon_{s} - \varepsilon_{\infty } } \right)\frac{2\pi f\tau }{{1 + \left( {2\pi f} \right)^{2} \tau^{2} }} $$

The relationships between the conductive and polarization loss of the various samples as functions of frequency are depicted in Fig. S9, respectively. Notably, the ε_p′′_ and ε_c′′_ values of the SiC@MoO_3_ nanocomposite remain stable with increasing frequency, maintaining low levels, and their combined impact on dielectric loss is negligible. This observation aligns with the trends depicted in Fig. 5a, b. Furthermore, as the frequency increases, the ε_c′′_ values of the various SiC@MO-t samples gradually decrease, indicating a diminishing contribution of conductive loss to the overall dielectric loss. The ε_c′′_ values of the SiC@MO-t4 sample consistently exceed those of other samples across all tested frequency ranges, indicating its pronounced conductive loss capability. The ε_c"_ values of SiC@MO-t2, SiC@MO-t6, and SiC@MO-t8 closely align, and given the notable differences in their electromagnetic wave absorption capabilities, it can be inferred that polarization loss plays a predominant role in mitigating the effects of incident electromagnetic waves on these samples. Figure S9b illustrates the polarization loss curves of each SiC@MO-t sample plotted against frequency, showing that the ε_p"_ values follow the trends depicted in the Fig. S9a. Notably, the polarization loss values in the SiC@MO-t2, SiC@MO-t6, and SiC@MO-t8 samples exceed their respective conduction loss values by a factor of one, confirming that polarization loss associated with oxygen vacancie predominates as the source of dielectric loss in these materials. Despite minimal difference in conduction loss values, the polarization loss values of the SiC@MO-t2 and SiC@MO-t8 samples are notably higher than that of the SiC@MO-t6 sample. This contrasts with the preceding results on reflection loss, implying that impedance matching associated with oxygen vacancies may exert a more significant influence on the enhancement of absorption performance than on dielectric loss performance. For the SiC@MO-t4 sample, in the S-band, its conduction loss values surpass its polarization loss values, whereas in the remaining measured bands, the contribution of polarization loss to dielectric loss is predominant over conduction loss. The conductivity (Fig. S10) of the SiC@MoO_3_ nanocomposite is notably lower than that of the SiC@MO-t samples containing oxygen vacancies, and its conductivity is correlated with the sequence of curves in Fig. 5b. This effect is likely attributed to oxygen vacancies that introduce defect levels into the band-gap of the MoO_3_ semiconductor, enhancing the migration and hopping capacity of surface electrons and thus resulting in significantly improved conductive loss characteristics [62].

To examine the polarization loss of the samples, the relaxation behavior of the various products was analyzed using Debye relaxation theory. The typical relationship between ε′ and ε′′ can be described by the following equation [45]:

10 $$ \left( {\varepsilon^{\prime} - \frac{{\left( {\varepsilon_{s} + \varepsilon_{\infty } } \right)}}{2}} \right)^{2} + \left( {\varepsilon^{\prime\prime}} \right)^{2} = \left( {\frac{{\varepsilon_{2} - \varepsilon_{\infty } }}{2}} \right)^{2} $$

Figure S11 exhibits the Cole–Cole curves of SiC@MoO_3_ nanocomposites. It can be observed that a single semicircle can be marked, suggesting that limited Debye relaxation phenomena occur, which cannot effectively dissipate the energy of the incident electromagnetic wave. The Cole–Cole plots of SiC@MoO_3_ nanocomposites and SiC@MO-t samples are depicted in Fig. 5d–g, respectively. It can be found that the introduction of oxygen vacancies in the SiC@MO-t sample results in additional semicircles, indicating a more pronounced Debye relaxation phenomenon relative to the SiC@MoO_3_ nanocomposites. Similar to the results presented in Fig. 5b, the SiC@MO-t4 and SiC@MO-t6 samples with optimal oxygen concentration, exhibit the most semicircles, indicating the strongest relaxation behavior among them and corresponding to better electromagnetic wave loss properties [63]. Furthermore, the long-tail phenomenon at the end of the Cole–Cole curve indicates the conduction loss of various samples, representing the conductivity loss contributing to electromagnetic wave attenuation of the absorber [64]. Analysis of the graph reveals that the calculated slope values of the samples are correlated with the relative magnitude of their conduction loss. Additionally, the different Cole–Cole curve tail slope values were calculated, as indicated by the blue line in Fig. 5h, revealing that SiC@MO-t4 sample exhibits strong conduction loss. It is well known that the excellent absorption performance of absorptive materials depends on two important parameters: impedance matching, which reflects the extent of electromagnetic waves penetrating the absorbing material, and the attenuation coefficient, which reflects the attenuation capacity of the absorber for incident electromagnetic waves. These two parameters may be calculated using the following formulas [65]:

11 $$ \alpha = \frac{\sqrt 2 \pi f}{{zc}}\sqrt {\left( {\mu^{\prime\prime}\varepsilon^{\prime\prime} - \mu^{\prime}\varepsilon^{\prime}} \right) + \sqrt {(\mu^{\prime\prime}\varepsilon^{\prime\prime} - \mu^{\prime}\varepsilon^{\prime})^{2} + (\mu^{\prime}\varepsilon^{\prime\prime} + \mu^{\prime\prime}\varepsilon^{\prime})^{2} } } $$

Figure 5i depicts the attenuation coefficient variations of the SiC@MoO_3_ nanocomposite and SiC@MO-t samples. Across the entire frequency range, the α values for each SiC@MO-t sample exceed those of SiC@MoO_3_ nanocomposite, with the relative positions of the curves correspond to those in Fig. 5b, which suggests a stronger attenuation capacity for the incident electromagnetic waves. These findings provide further evidence that oxygen vacancies promote electromagnetic wave loss, consistent with the observations from the polarization loss curve shown in Fig. S9. The maximum value for the SiC@MoO_3_ nanocomposite is observed at approximately 6 GHz, reaching nearly 100. In other examined frequency ranges, the values are relatively small and exhibit negligible variation, indicating a poor attenuation capability against incident electromagnetic waves. In the S-band, the attenuation coefficient values for each SiC@MO-t sample appear to be comparatively uniform. In the C and X bands, the attenuation coefficient values for each SiC@MO-t sample demonstrate a gradual increase with respect to frequency. In the Ku frequency band, the attenuation coefficient values for each SiC@MO-t sample tend to fluctuate in proximity to their maximum values. The delta function (|Δ|) was computed to evaluate the impedance matching of SiC@MoO_3_ nanocomposite with various SiC@MO-t samples with the following equation [66]:

12 $$\mid\Delta \mid =\mid {\text{sinh}}^{2}\left(\text{k}\widehat{\text{f}}\text{d}\right)-\text{M}\mid $$

The constants K and M can be determined from the relationship between complex permittivity and complex permeability, as indicated in the following equation:

13 $$\text{K}=\frac{4\uppi \sqrt{{\upmu }_{\text{r}}{\upvarepsilon }_{\text{r}}}\text{sin}\left(\frac{{\updelta }_{\text{e}}+{\updelta }_{\text{m}}}{2}\right)}{\text{ccos}{\updelta }_{\text{e}}\text{cos}{\updelta }_{\text{m}}}$$

14 $$ M = \frac{{4\mu^{\prime}\cos \delta_{\varepsilon }{\prime} \cos \delta_{m} }}{{(\mu^{\prime}\cos \delta_{\varepsilon } - \varepsilon^{\prime}\cos \delta_{m} )^{2} + [\tan \left( {\frac{{\delta_{m} - \delta_{\varepsilon } }}{2}} \right)]^{2} (\mu !\cos \delta_{\varepsilon } + \varepsilon^{\prime}\cos \delta_{m} )^{2} }} $$

Optimal impedance matching in the absorbers is achieved when the absolute value of |Δ| approaches zero. Effective impedance matching for electromagnetic wave absorbing materials occurs when |Δ| falls below 0.4. Figure 5j–m depicts the |Δ| values for a range of samples under optimal conditions. Notably, the SiC@MO-t4 and SiC@MO-t6 samples exhibit a broader range where |Δ| is less than 0.4, suggesting enhanced impedance matching that enables incident electromagnetic waves to penetrate the material more effectively. This indicates that while excellent attenuation capacity is crucial for superior electromagnetic loss performance, combing it with excellent impedance matching is equally fundamental [67]. Based on the aforementioned evidence, one can conclude that oxygen vacancies have a significant impact on both the attenuation coefficient and the impedance matching characteristics of SiC@MoO_3_ nanocomposite.

### Electromagnetic Wave Attenuation Mechanism of Oxygen Vacancy-Rich SiC@MoO_3_ Nanocomposites

**Fig. 6:**
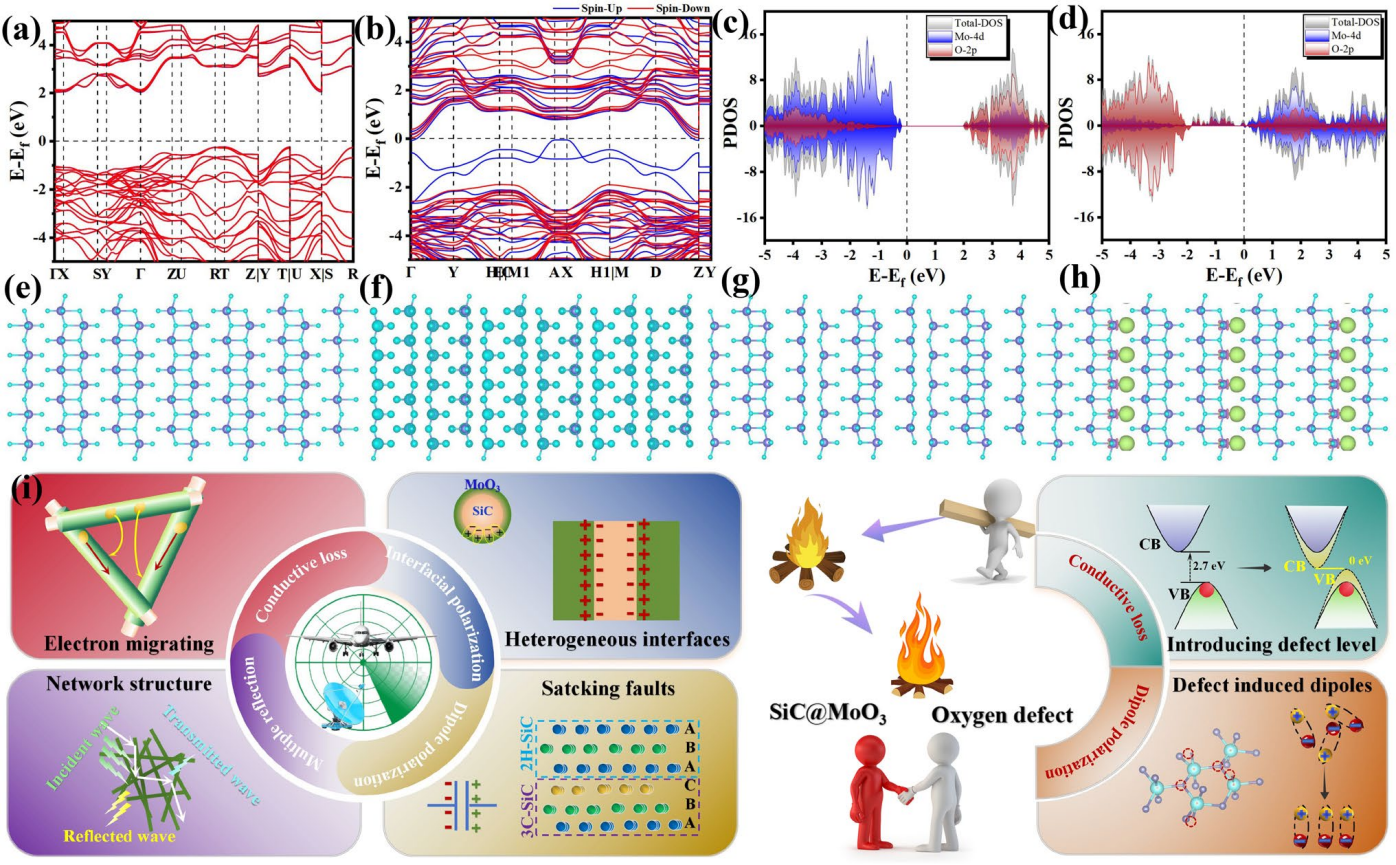
Energy structure of **a** SiC@MoO_3_ nanocomposite and **b** SiC@MO-t sample. Projected density of state of **c** SiC@MoO_3_ nanocomposite and **d** SiC@MO-t sample. **e** Atomic structure and **f** charge distribution of SiC@MoO_3_ nanocomposite. **g** Atomic structure and **h** differential charge density of SiC@MO-t sample. **i** Schematic diagram of electromagnetic wave attenuation mechanism

To comprehensively understand the impact of oxygen vacancies on electromagnetic wave absorption, the electronic distribution around these vacancies in various samples was investigated using density functional theory (DFT) calculations. Since identical SiC nanowires acted as carriers or substrate, simplifying the model, the alterations in the electronic structure of MoO_3_ across different samples were investigated. Figure 6a–d depicts atomic structure models and band structures of intrinsic MoO_3_ and MoO_3_ with oxygen vacancies. It is well known that in semiconductor materials, oxygen vacancies (V_O_) can reduce the hybridization energy level, thereby decreasing the band gap [68]. Under the influence of oxygen vacancies, neighboring electrons around the low-coordination Mo atoms enter the conduction band, thereby increasing the conductivity. Consequently, the band gap of MoO_3_ in the SiC@MO-t sample decreases from the direct band gap of intrinsic MoO_3_ with the value of 2.27 to 0 eV. This narrowing facilitates electron transfer and hopping, enhancing the conductivity loss effect of electromagnetic wave [69]. Therefore, compared to SiC@MoO_3_ nanocomposites, the conductivity loss of SiC@MO-t samples is significantly enhanced.

Figure 6c, d depicts the density of states (DOS) for bare MoO_3_ and MoO_3_ with oxygen vacancies, respectively. For samples containing oxygen vacancies, it can be observed that the valence band maximum is mainly composed of O-2*p* orbitals. In the energy region higher than the O-2*p* orbitals, some new Mo-4*d* and O-2*p* orbitals appear, indicating that oxygen vacancies cause additional Mo-4*d* and O-2*p* electronic orbitals, resulting in a narrowing the band gap of system. From Fig. 6e, f, it can be seen that for intrinsic MoO_3_, the charge density distribution shows no significant differences across the entire model. On the other hand, Fig. 6g, h displays the charge difference for MoO_3_ with oxygen vacancies, clearly indicating charge transfer at the positions of missing oxygen atoms. This implies that the absence of lattice oxygen disrupts the symmetric charge distribution, leading to the induction of electric dipoles and the formation of positive charge centers, which act as enhanced dipole polarization in alternating electromagnetic fields, contributing to the attenuation of incident electromagnetic waves [20].

Based on the above analysis, the SiC@MO-t sample can be regarded as a nanocomposite consisting of SiC@MoO_3_ coupled with oxygen vacancy defects. The impact of SiC@MO-t on incident electromagnetic waves results from the combined dielectric loss performances. For the SiC@MoO_3_ nanocomposite, the mechanism diagram illustrating its role in attenuating electromagnetic waves is shown in Fig. 6i. The main mechanism can be summarized as follows: (1) Structurally, the densely packed SiC@MoO_3_ one-dimensional nanomaterials form an interwoven network. The disordered pore structure, surrounded by adjacent nanowires, functions as a transmission channel for electromagnetic waves to penetrate into the interior of the network. Simultaneously, it enhances multiple reflections and scattering of electromagnetic waves, effectively improving the dielectric loss capacity of the product [70]. (2) While both β-SiC and α-MoO_3_ are wide band-gap semiconductor materials, the band-gap of latter is approximately 2.72 eV, smaller than that of the former, which has a value of 3.13 eV. Compared to single SiC nanowires, the synthesized SiC@MoO_3_ one-dimensional nanomaterials exhibit a narrower band-gap [71, 72]. This reduction diminishes the energy barriers for electron migration and hopping, potentially enhancing the electrical loss characteristics of the sample to a certain extent. (3) Due to the significant difference in electron transfer efficiency between SiC and MoO_3_, charge carriers may become trapped at the hetero-junction interface during migration. This leads to the accumulation and non-uniform distribution of charges around the interface region, which in turn generates abundant interface polarization losses [73]. (4) Within SiC nanowire structures, stacking faults, along with atoms in the perfect crystal lattice, may serve as polarization centers. This induces the redistribution of positive and negative charges, creating imbalances in dipole moments and subsequent dipole polarization formation [25, 70]. This mechanism further contributes to enhanced dielectric loss properties of the product. Oxygen vacancy defects in the SiC@MoO_3_ sample play a crucial role in enhancing its absorption performance compared to the SiC@MoO_3_ nanocomposite. Firstly, oxygen vacancies modify the intrinsic band structure of MoO_3_ semiconductors by introducing defect energy levels, thereby reducing the energy barrier for electron transitions [74]. This enhancement in electron transport results in stronger electrical loss capabilities relative to SiC@MoO_3_ nanocomposite. Furthermore, the introduction of oxygen vacancies in the MoO_3_ lattice induces lattice distortion and serves as polarization centers. These vacancies capture and release charge carriers, altering the charge distribution around them and generating strong defect-induced dipole moments [12]. This emphasizes the role of polarization loss in attenuating electromagnetic waves. Moreover, the presence of oxygen vacancies facilitates the tuning of impedance matching between the SiC@MO-t sample and its surrounding environment, enabling enhanced penetration of electromagnetic waves that impinge on the surface of the material into the absorber. This optimizes the dissipation of electromagnetic wave energy.

**Fig. 7:**
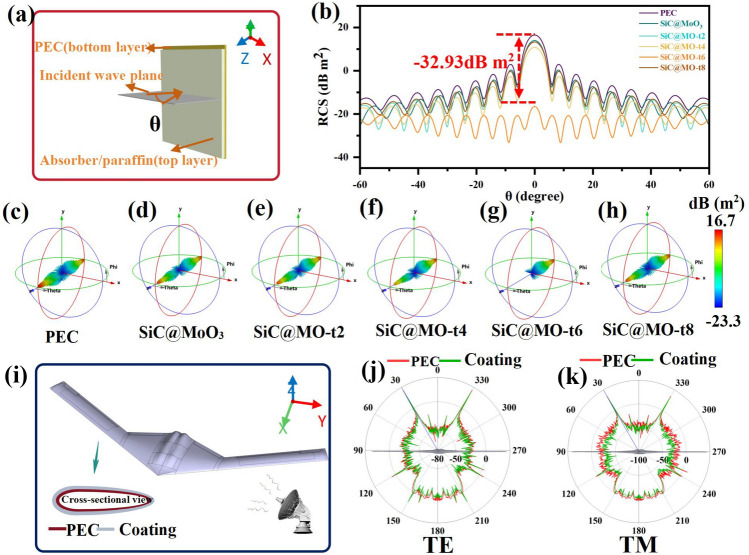
**a** Theta polarization model, **b** RCS simulation curves of PEC in the test range of − 60.0° < θ ≤ 60.0° for SiC@MO-t6. **c-h** CST far-field simulation results of PEC, SiC@MoO_3_, SiC@MO-t2, SiC@MO-t4, SiC@MO-t6, and SiC@MO-t8. **i** Drone model with ultra-wideband electromagnetic wave absorption performance. **j, k** RCS curves under TE polarization and TM polarization

To demonstrate the effectiveness of SiC@MoO_3_ nanocomposite and SiC@MO-t samples, which are rich in oxygen vacancies, in broadband and wide-angle electromagnetic wave absorption, this study also aims to verify the stealth performance of the absorbers in practical applications. Under the detection frequency of 14.96 GHz, 3D far-field simulation maps of the samples were perfectly simulated (Fig. 7c–h). Figure 7a shows the establishment of a perfect PEC plate model. Figure 7b shows the PEC model and the maximum RCS value of SiC@MO-t sample as − 32.93 dB m^2^. Generally, the radar cross-section (RCS) value of a material represents the physical quantity of the F-reflected wave intensity detected by radar under aircraft coating conditions, and a smaller RCS value indicates better stealth performance of the material. The RCS of the aircraft is related to the unit solid angle reflection in the radar receiving direction [75]. Additionally, the basic equation describing the radar and the radar intercept signal value, as well as the power density of target firing, are relevant. The equation for calculating the cross-section (RCS, σ) can be expressed as [76]:

15 $$\sigma \left({m}^{2}\right)=\underset{R\to \infty }{\text{lim}}4\pi {R}^{2}{\left(\left|\frac{{E}_{s}}{{E}_{i}}\right|\right)}^{2}=\underset{R\to \infty }{\text{lim}}4\pi {R}^{2}{\left(\left|\frac{{H}_{s}}{{H}_{i}}\right|\right)}^{2}=\underset{R\to \infty }{\text{lim}}4\pi {R}^{2}\frac{{S}_{s}}{{S}_{i}}$$

In the formula, *E*_s_ and *E*_i_ represent the intensity of scattered electric field and incident electric field respectively. *H*_s_ and *H*_i_ represent the intensity of scattering c magnetic field and incident magnetic field respectively. S_s_ and S_i_ represent the power densities of scattering and incident lengths. Accordingly, a 3D far-field simulation was conducted at a detection frequency of 14.96 GHz for five materials. Since kα is greater than 10, and the target size of the aircraft model is larger than the signal wavelength [77]. In addition, the forward (Fig. 7j) and top-down (Fig. 7k) RCS (measuring the transverse electropolarized waves of the returned sensor) of the UAV at the optimal frequency (14.96 GHz) show that the composite structure reflects a weaker electromagnetic wave signal than the basic PEC model. The results show that SiC@MO-t series absorbing materials rich in oxygen vacancies have excellent electromagnetic wave absorption capability, and exhibit excellent attenuation strategy to effectively regulate the radio wave absorption under complex conditions.

## Conclusion

In this study, SiC@MoO_3_ nanocomposites with controllable vacancy concentration were synthesized by the in situ growth of MoO_3_ on the surface of pre-obtained SiC nanowires, followed by reduction using KBH_4_. Characterization results indicate that the presence of oxygen vacancies directly enhances dipole polarization, thereby improving the effectiveness of electromagnetic loss. Analysis reveals that the optimized SiC@MO-t4 nanocomposite exhibits superior electromagnetic wave absorption performance, achieving an *RL*_min_ value of -50.49 dB at a matching thickness of 1.27 mm. The SiC@MO-t6 nanocomposite exhibits exceptional performance, with an impressive EAB of 8.72 GHz at a matching thickness of 2.81 mm, covering the entire Ku band. Remarkably, both nanocomposites outperform the SiC@MoO_3_ sample in terms of electromagnetic loss efficiency. Furthermore, the exploitation of vacancy-induced direct dipole polarization presents a promising approach for enhancing electromagnetic wave absorption capabilities through defect engineering. This strategy holds potential applications in the development and enhanced performance of other absorbers.

## Supplementary Information

Below is the link to the electronic supplementary material.

Supplementary file 1 (DOCX 1531 KB)
