# Supplementary material for: Enhancing Defect-Induced Dipole Polarization Strategy of SiC@MoO3 Nanocomposite Towards Electromagnetic Wave Absorption
Source: Nanomicro Lett. 2024 Aug 16;16:273. doi: 10.1007/s40820-024-01478-2 (PMC11327238; doi:10.1007/s40820-024-01478-2)
Supplement: Supplementary file 1 — (DOCX 1531 KB) [file 40820_2024_1478_MOESM1_ESM.docx]

# Supporting Information for

Enhancing Defect-Induced Dipole Polarization Strategy of SiC@MoO_3_ Nanocomposite Towards Electromagnetic Wave Absorption

Ting Wang^1^, Wenxin Zhao^2^, Yukun Miao^2^, Anguo Cui^3^, Chuanhui Gao^1^, Chang Wang^2^, Liying Yuan^2^, Zhongning Tian^2^, Alan Meng^4^, Zhenjiang Li^2,^ *, Meng Zhang^2,^ *

^1^ College of Chemical Engineering, Qingdao University of Science and Technology, Qingdao, Shandong province, 266042, P. R. China

^2^ College of Materials Science and Engineering, Qingdao University of Science and Technology, Qingdao, Shandong province, 266042, P. R. China

^3^ Shandong Engineering Laboratory for Preparation and Application of High-Performance Carbon-Materials, College of Electromechanical Engineering, Qingdao University of Science and Technology, Qingdao 266061, P. R. China

^4^ Key Laboratory of Optic-electric Sensing and Analytical Chemistry for Life Science, MOE, Shandong Key Laboratory of Biochemical Analysis, College of Chemistry and Molecular Engineering, Qingdao University of Science and Technology, Qingdao, Shandong province, 266042, P. R. China

*Corresponding authors. E-mail: [mengzhang@qust.edu.cn](mailto:mengzhang@qust.edu.cn) (Meng Zhang); [zjli126@126.com](mailto:zjli126@126.com) (Zhenjiang Li)

**Supplementary Figures and Table**


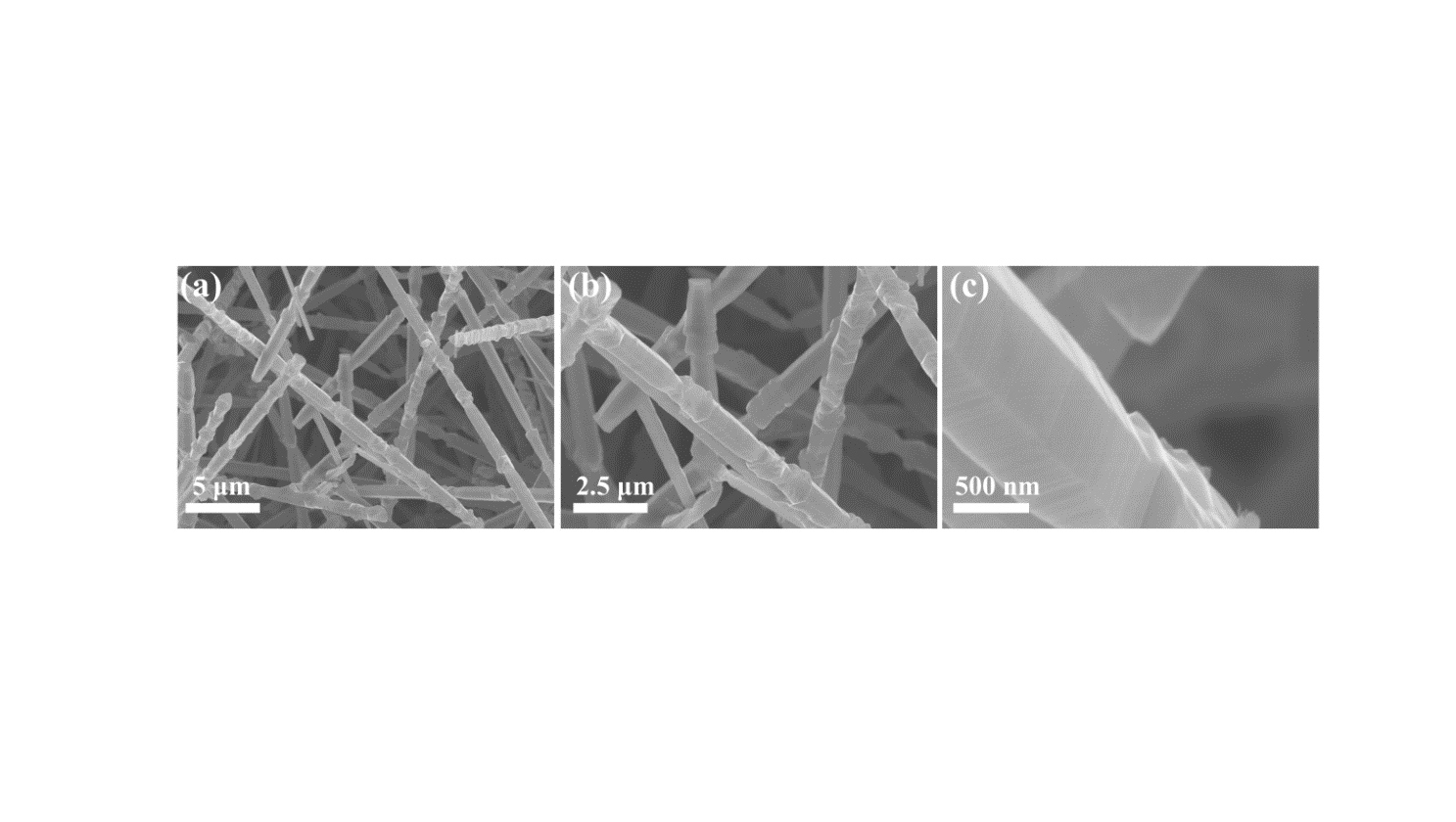


**Fig. S1** **a~c** SEM images of SiC_NWS_

**Fig. S2** EDX spectrum of SiC@MoO_3_ nanocomposite


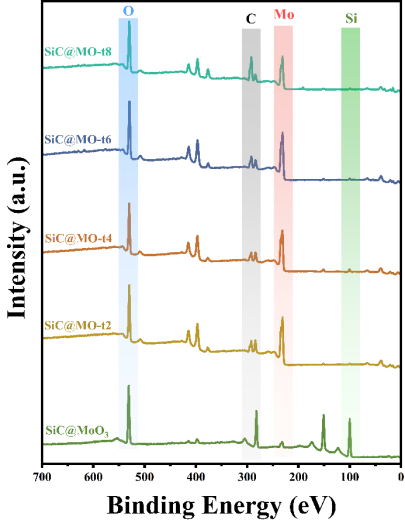

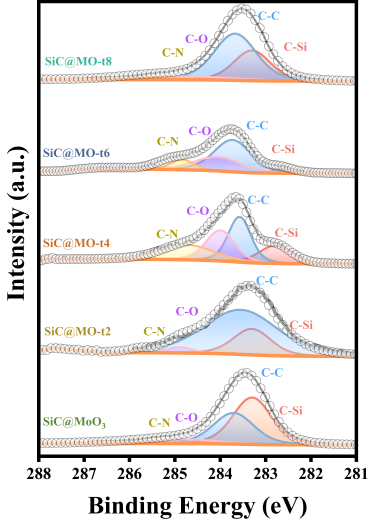

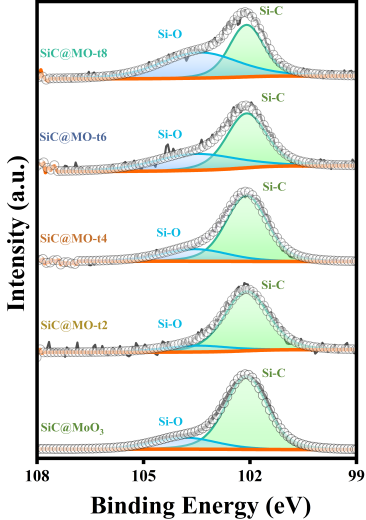


(a)

(c)

(b)

**Fig. S3** **a** XPS spectra full spectrum, **b** C1s and **c** Si 2p spectra of SiC@MoO_3_, SiC@MO-t2, SiC@MO-t4, SiC@MO-t6 and SiC@MO-t8


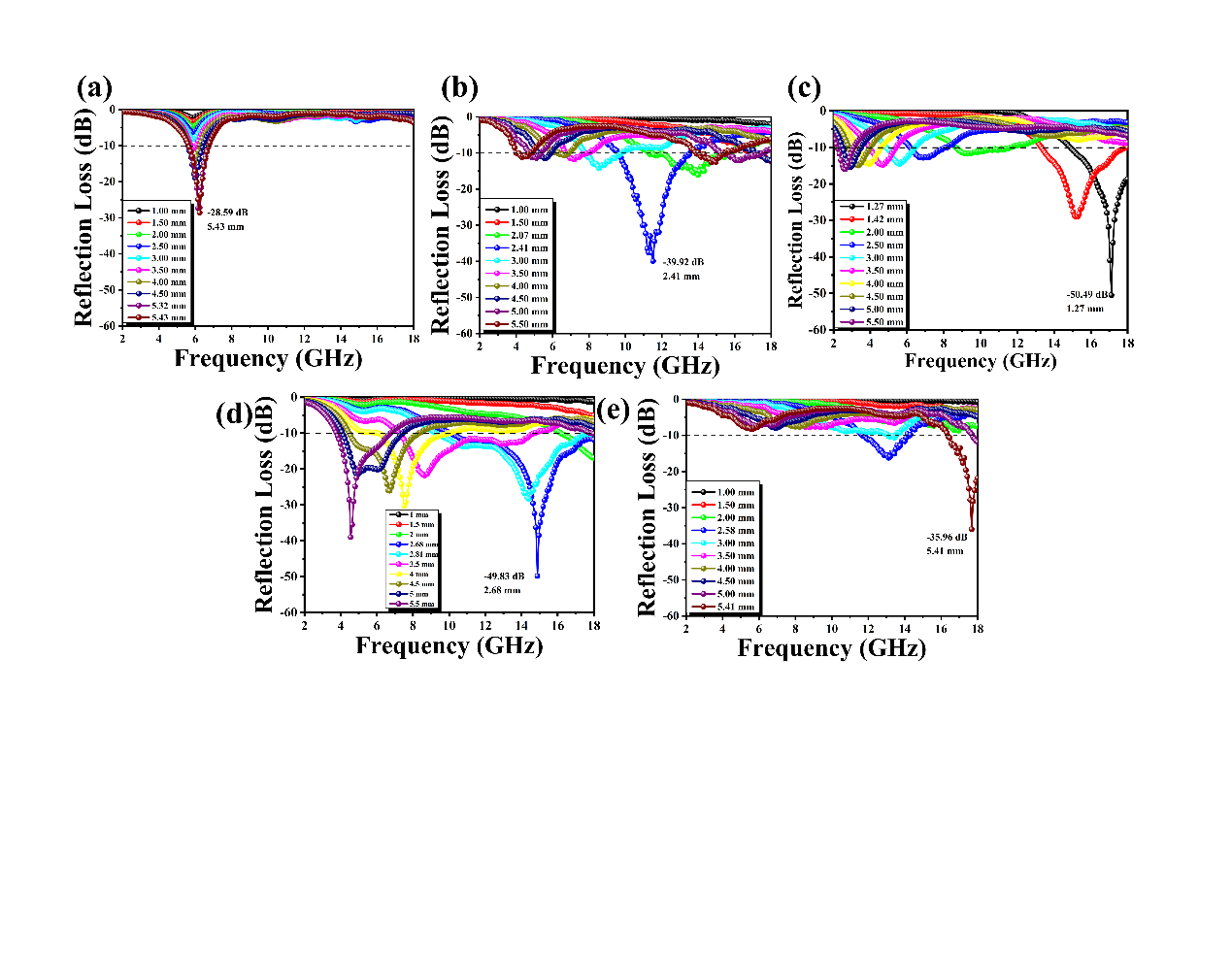


**Fig. S4** Two-dimensional graph of **a** SiC@MoO_3_, **b** SiC@MO-t2, **c** SiC@MO-t4, **d** SiC@MO-t6 and **e** SiC@MO-t8


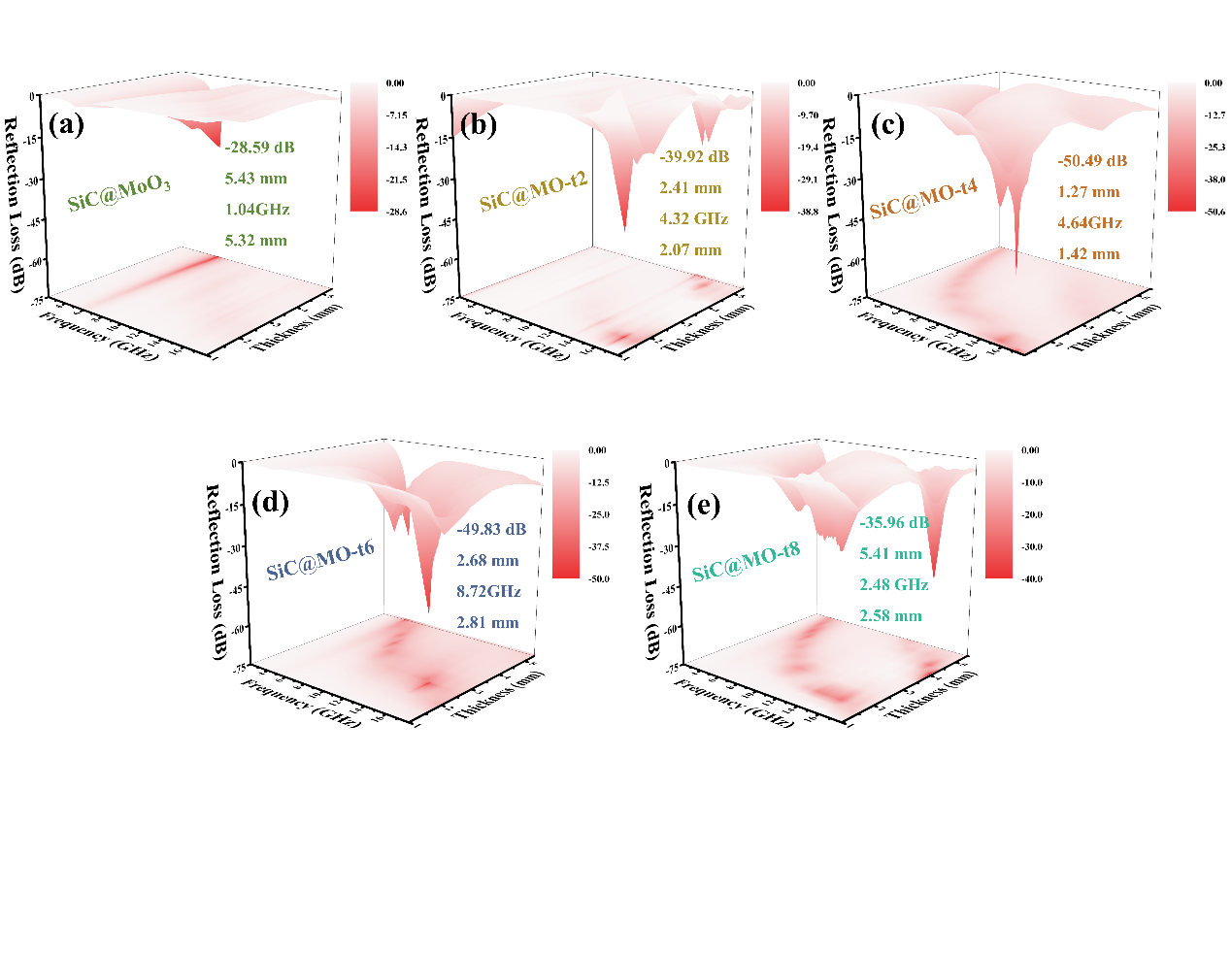


**Fig. S5** Three-dimensional graph of **a** SiC@MoO_3_, **b** SiC@MO-t2, **c** SiC@MO-t4, **d** SiC@MO-t6 and **e** SiC@MO-t8


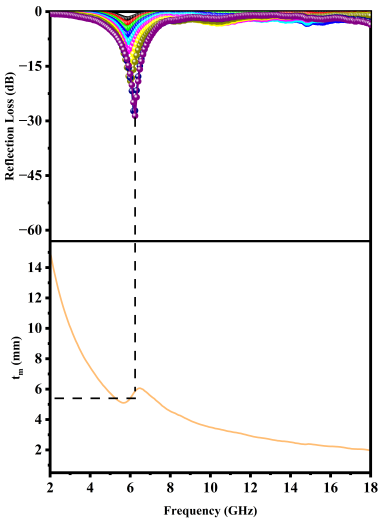

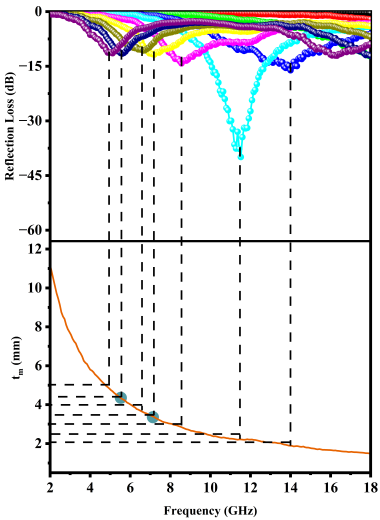

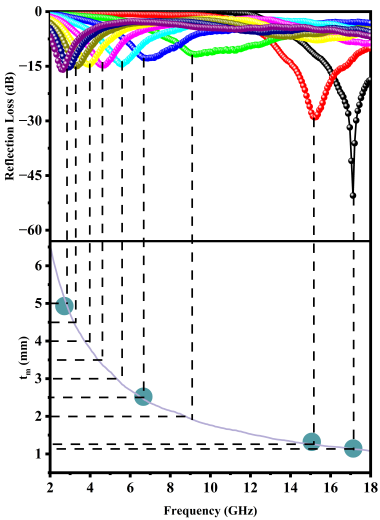

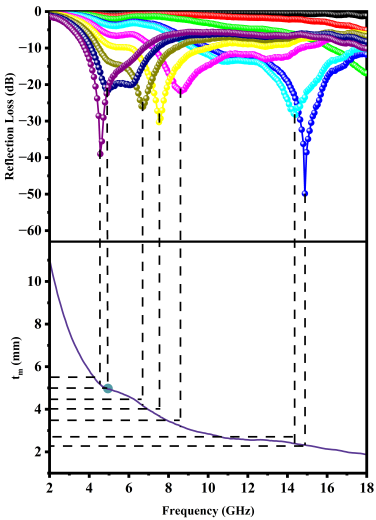

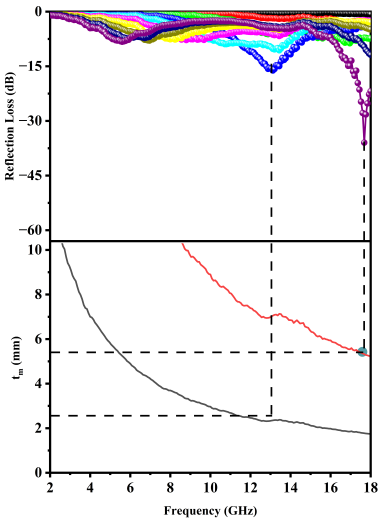


(e)

(d)

(c)

(b)

(a)

**Fig. S6** Absorption curves and corresponding 1/4 λ matching properties of **a** SiC@MoO_3_ nanocomposite and **b** SiC@MO-t2, **c** SiC@MO-t4, **d** SiC@MO-t6 and **e** SiC@MO-t8


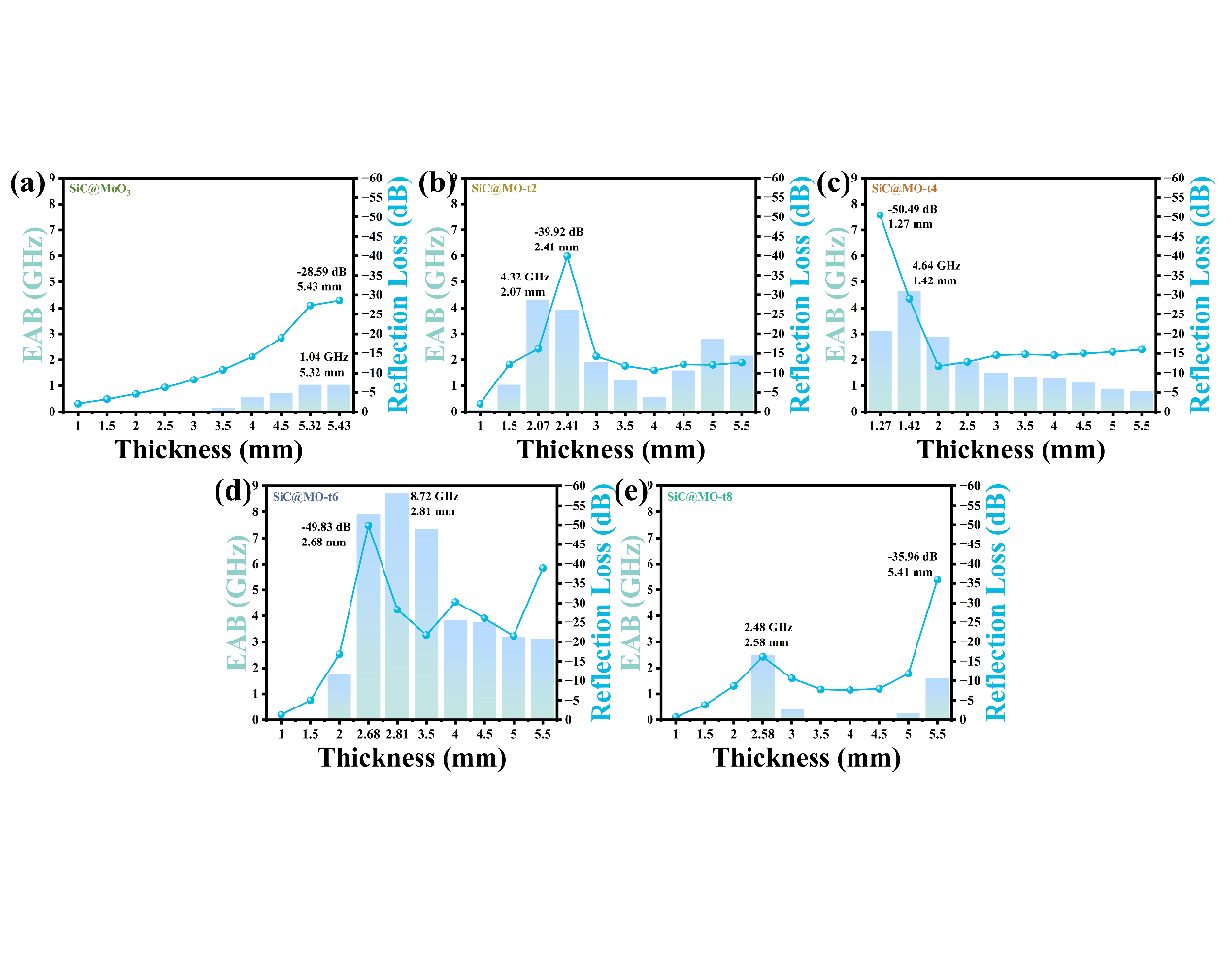


**Fig. S7** EAB and RL_min_ values of different samples under different matching thicknesses **a** SiC@MoO_3_ nanocomposite, **b** SiC@MO-t2, **c** SiC@MO-t4, **d** SiC@MO-t6 and **e** SiC@MO-t8


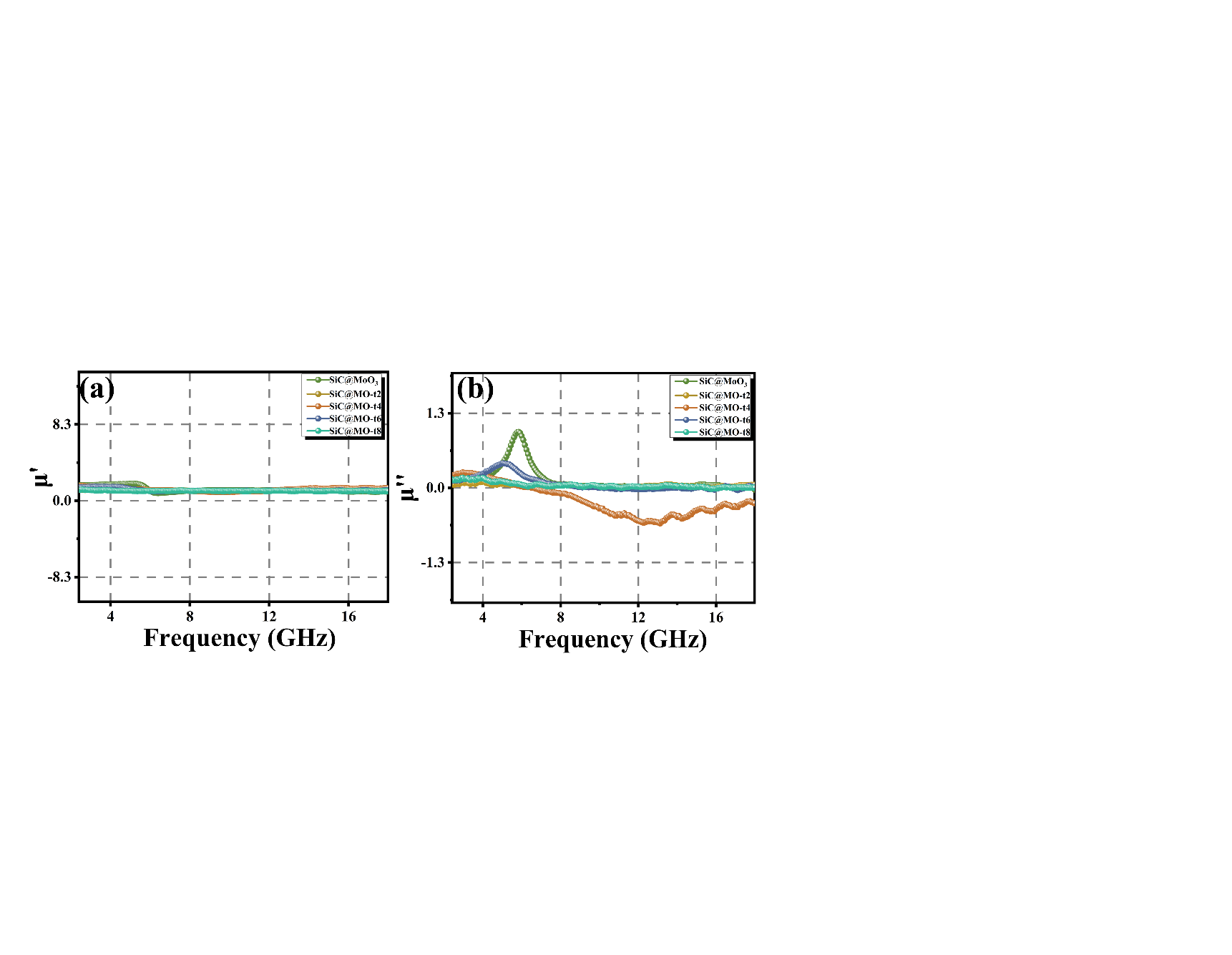


**Fig. S8 a** Magnetic permeability real and **b** magnetic permeability imaginary part of SiC@MoO_3_, SiC@MO-t2, SiC@MO-t4, SiC@MO-t6 and SiC@MO-t8


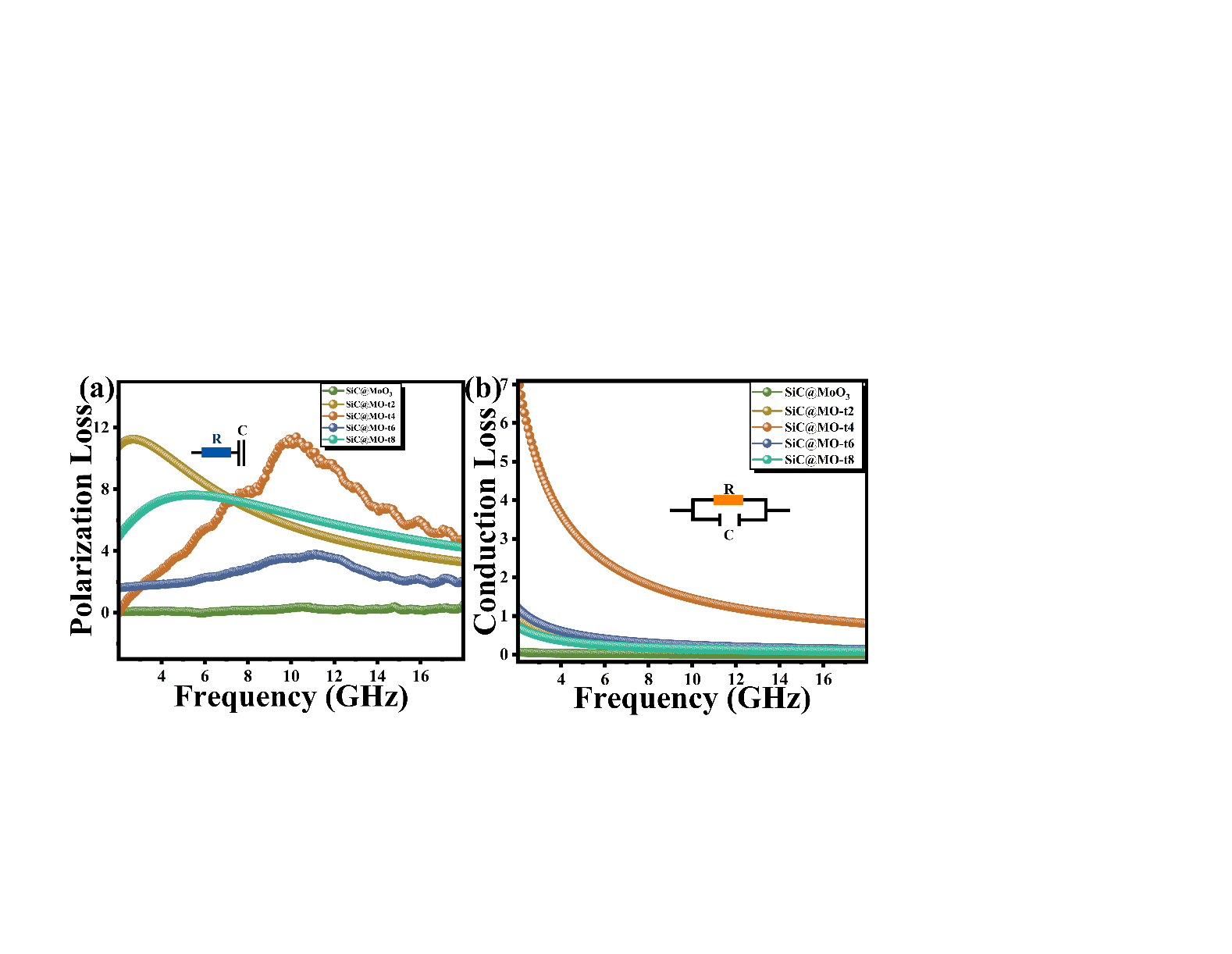


**Fig. S9** **a** Polarization Loss and **b** Conduction Loss values of SiC@MoO_3_, SiC@MO-t2, SiC@MO-t4, SiC@MO-t6 and SiC@MO-t8


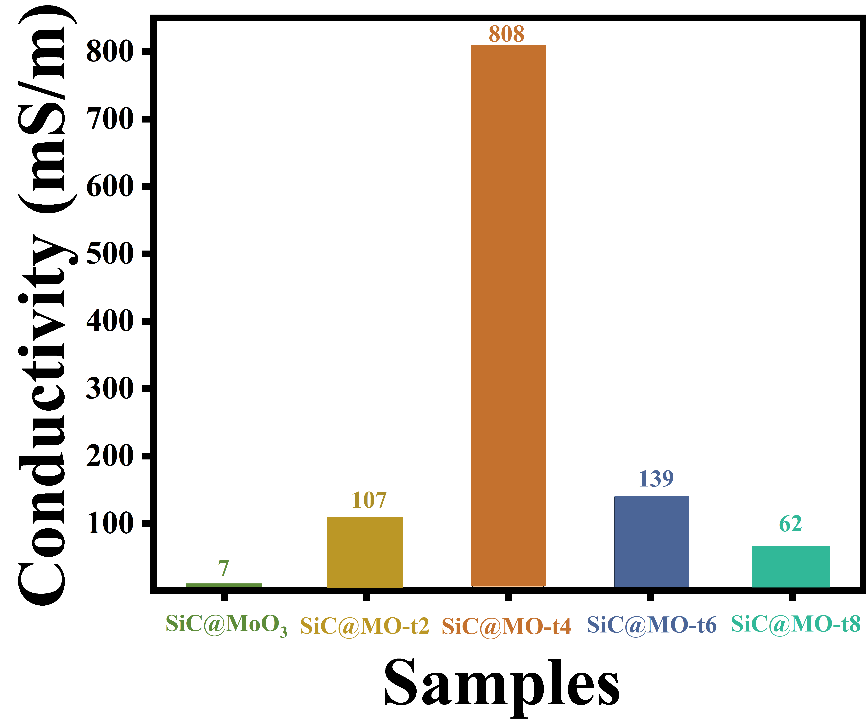


**Fig. S10** Conductivity values of SiC@MoO_3_, SiC@MO-t2, SiC@MO-t4, SiC@MO-t6 and SiC@MO-t8


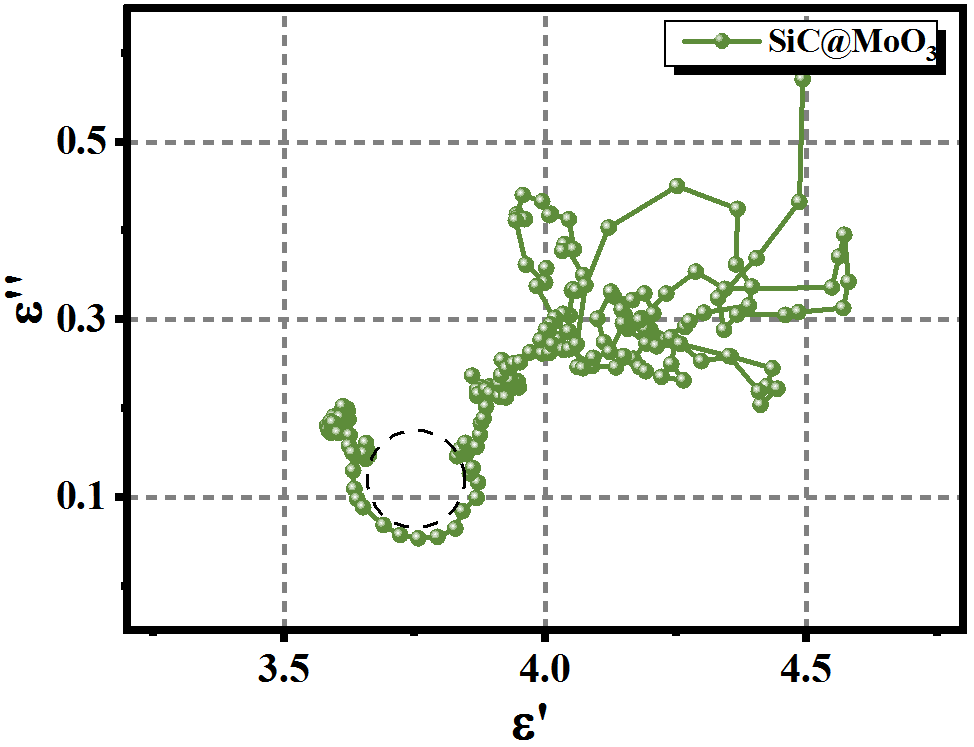


**Fig. S11** Cole-Cole curve of SiC@MoO_3_ nanocomposites

**Table R1** Comparison of RLmin and EAB values of the SiC@MO-t4 and SiC@MO-t6 samples with recent reports related absorbers

| Samples | EAB  (GHz) | Matching thickness (mm) | RL_min_  (dB) | Matching thickness (mm) | References |
| --- | --- | --- | --- | --- | --- |
| o-MoO_3_/PPy | 6.40 | 2.30 | -31.80 | 2.30 | [45] |
| MoO_3_/TiO_2_/Mo_2_TiC_2_T_x_ | 8.60 | 1.80 | -30.76 | 2.30 | [46] |
| graphene/g-C_3_N_4_ | 4.60 | 4.50 | -34.69 | 4.50 | [47] |
| SnO/SnO_2_ | 4.3 | 1.40 | -37.60 | 1.40 | [48] |
| Fe_3_O_4_/Fe_3_S_4_ | 4.39 | 1.46 | −45.33 | 3.30 | [49] |
| Fe_3_O_4_@SiO_2_@MnO_2_ | 5.1 | 2.60 | -50.20 | 2.60 | [50] |
| Fe_3_O_4_@MnO_2_@Ni–Co/C | 7.1 | 2.00 | -41.2 | 4.00 | [51] |
| ZnFe_2_O_4_@MnO_2_@MXene | 4.7 | 1.40 | -50.4 | 1.40 | [52] |
| **SiC@MO-t4** | **4.64** | **1.42** | **-50.49** | **1.27** | **This work** |
| **SiC@MO-t6** | **8.72** | **2.81** | **-49.83** | **2.68** | **This work** |
